# Diapause vs. reproductive programs: transcriptional phenotypes in a keystone copepod

**DOI:** 10.1038/s42003-021-01946-0

**Published:** 2021-03-29

**Authors:** Petra H. Lenz, Vittoria Roncalli, Matthew C. Cieslak, Ann M. Tarrant, Ann M. Castelfranco, Daniel K. Hartline

**Affiliations:** 1 Pacific Biosciences Research Center, University of Hawaiʻi at Mānoa, Honolulu, HI USA; 2 Stazione Zoologica Anton Dohrn, Napoli, Italy; 3 Woods Hole Oceanographic Institution, Woods Hole, MA USA

**Keywords:** Ecophysiology, Molecular ecology, Ecology, Molecular biology, Transcriptomics

## Abstract

Many arthropods undergo a seasonal dormancy termed “diapause” to optimize timing of reproduction in highly seasonal environments. In the North Atlantic, the copepod *Calanus finmarchicus* completes one to three generations annually with some individuals maturing into adults, while others interrupt their development to enter diapause. It is unknown which, why and when individuals enter the diapause program. Transcriptomic data from copepods on known programs were analyzed using dimensionality reduction of gene expression and functional analyses to identify program-specific genes and biological processes. These analyses elucidated physiological differences and established protocols that distinguish between programs. Differences in gene expression were associated with maturation of individuals on the reproductive program, while those on the diapause program showed little change over time. Only two of six filters effectively separated copepods by developmental program. The first one included all genes annotated to RNA metabolism and this was confirmed using differential gene expression analysis. The second filter identified 54 differentially expressed genes that were consistently up-regulated in individuals on the diapause program in comparison with those on the reproductive program. Annotated to oogenesis, RNA metabolism and fatty acid biosynthesis, these genes are both indicators for diapause preparation and good candidates for functional studies.

## Abstract (web summary)

Lenz, Roncalli and colleagues examine the difference in transcriptional phenotype in *Calanus finmarchicus* during reproductive and dormant phases. Their findings indicate gene targets for assessment of reproductive strategies in this high latitude copepod crucial to marine food webs.

## Introduction

Large copepods are at the base of the metazoan food web of high-latitude marine ecosystems that support highly productive fisheries^1–3^. Low recruitment of young-of-the-year fish larvae in the North Atlantic, North Pacific and the Bering Sea have been correlated with below-average abundances of these lipid-rich copepods^4–8^. While their population abundances do correlate negatively with temperature^5,9^, observed temperatures are well within known species’ tolerances^10^, suggesting that indirect effects may be more important. Changes in ocean circulation patterns and the timing and magnitude of spring phytoplankton blooms could have major impacts on zooplankton abundances and distributions^11–13^. Furthermore, lipid-rich copepods have complex life histories and depend on a seasonal dormancy (diapause) to ensure the continued presence of a strong spring population in a system. Thus, poor spring recruitment due to changes in diapause could be a tipping point for a local ecosystem. However, the copepods’ adaptive potential and phenotypic plasticity are unknown and require a more precise understanding of diapause and how it is controlled before environmental tipping-points can be predicted.

Our current understanding of the life cycle and ecology of lipid-rich copepods has emerged mostly from studies on *Calanus finmarchicus*, a keystone species that plays a central role in North Atlantic food webs^2,4,14–16^. Its annual cycle begins with generation G0 when copepods emerge from diapause as pre-adults (copepodid stage CV), molt into adults, mate, and reproduce^17,18^. The offspring (G1) of the G0 generation then make a critical “choice”: some individuals develop directly through six naupliar and six copepodid stages into adults (“reproductive program”) and produce another generation (G2), while others develop to the CV stage, then migrate to depth and enter diapause (“diapause program”)^14,19–21^. In the Gulf of Maine, *C. finmarchicus* can complete up to three such generations annually, with each generation in turn appearing to contribute to the overwintering population^19,22^. In contrast, those of very high latitudes, such as the Norwegian Sea, have only one (G1) generation per year; all are on the diapause program^23^. Copepods destined to diapause accumulate lipids that fuel the dormant period and contribute energetically to reproduction post-diapause^17,24^.

Predicted changes in phenology in response to ocean warming^25^ raise two central questions about diapause in *C. finmarchicus*. The first one, how many, which and when copepods from each generation initiate the diapause program, is critical for assessing recruitment in the following year and for predicting the population cycle in the current year. Copepods that migrate to depth take their accumulated lipids with them and thus reduce lipid availability for upper trophic levels in surface waters. Those entering into diapause sequester biomass and lipids, effectively removing carbon at least temporarily from the upper 100 m and placing it into long-term storage for later availability to the ecosystem^15,26–28^. The second question relates to the basic biology and evolution of post-embryonic diapause in copepods. Developmental programs that include dormancy have evolved multiple times in arthropods^29^. The copepod diapause differs in that it is unlikely to be regulated by temperature and/or photoperiod^14^. A central question then is how does the copepod diapause compare with that of other organisms? What physiological characteristics are shared and which ones differ? While depressed metabolic rates and an arrest in development characterize diapause^24,30^, gene expression studies suggest that the specific molecular mechanisms that control diapause vary among taxa^29,31–34^. Modern transcriptomic technology permits examining a comprehensive array of genes involved in all aspects of an organism’s life, and thus offers an opportunity to address both questions.

The inability to distinguish between uninterrupted (reproductive program) vs. interrupted (diapause program) life-history phenotypes is an impediment to a mechanistic understanding of how the decision to enter the diapause program changes depending on genotype, environment, and season. While major programmatic differences in physiology have been demonstrated in insects, these studies have relied on controlled experimental conditions^35–37^. In contrast, developmental program is difficult to assess in field-collected individuals of species with facultative diapause and unknown triggers for entering the diapause program. This includes *C. finmarchicus*^23,24^. However, once program-specific patterns in gene expression have been characterized, the how-when-why of diapause initiation can be investigated. A transcriptomic approach that reliably distinguishes reproductive-program from diapause-program stage CV individuals could transform *C. finmarchicus* population studies by enabling tracking of how the number (and proportion) of diapause-program CVs changes during the season.

### Analysis strategy for an existing RNA-Seq dataset

Our goal was to determine whether the two programs could be separated by their respective gene expression (transcriptomic) phenotypes and whether this difference would lead to new insights into the physiological basis of the diapause program. The analysis was based on an RNA-Seq dataset generated by Tarrant and colleagues that included predominantly *C. finmarchicus* pre-adult copepodid stage CVs on different developmental programs^23,38^. These data allowed a broad-based comparison of transcriptional profiles of copepods on either the reproductive or the diapause program.

The RNA-Seq dataset comprised two-time points each, obtained from two sources of copepod: a laboratory-cultured group that was on the reproductive program, and a field-collected group from Trondheimsfjord that was on the diapause program. On close examination, the latter group was discovered to contain a limited admixture of two additional congeners, also on the diapause program, which we found had little impact on the results (see “Methods” and details in the Supplementary Note). The laboratory culture had been originally isolated from the same local fjord^39^. The laboratory-culture experimental groups were based on the number of days after molting into copepodid stage CV. Such history was unknown for the field copepodids. The analysis was tailored to identify gene expression differences that could be linked to the diapause program with the goal of excluding culture vs. field effects, or differences related to maturation within the molt-cycle.

**Fig. 1:**
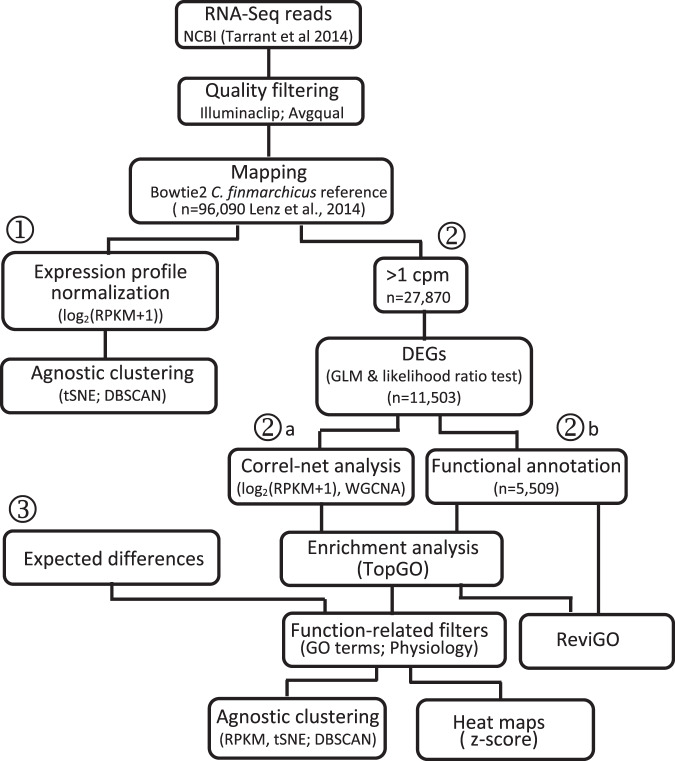
Diagram of workflow. Three different strategies used to assess the physiological ecology of copepods in the different samples are laid out using circled numbers. Initial steps included downloading of RNA-Seq data, removal of low-quality reads and sequence trimming followed by mapping against the Gulf of Maine *Calanus finmarchicus* annotated reference transcriptome (96 K transcripts) using Bowtie2. The mapped count data were normalized and log-transformed before dimensionality reduction by t-SNE and identification of clusters using DBSCAN (strategy 1). For differential gene expression analysis (DEGs), the mapped count data were entered into EdgeR for statistical testing using a generalized linear model (GLM) (strategy 2). The downstream analysis involved weighted correlation network analysis (WGCNA) on the log-transformed expression of the DEGs (sub-strategy 2a). SwissProt-based annotations for the DEGs were retrieved from the reference transcriptome and distribution of DEG function was visualized using ReviGO (sub-strategy 2b). DEGs from the GLM analysis and WGCNA modules were assessed for enriched processes (TopGO). Enrichment results in combination with expected differences in physiology were used to generate GO filters and retrieve log-transformed relative expression of all genes in the reference annotated to a specific filter for additional t-SNE and DBSCAN analyses (strategy 3). Gene expression patterns were visualized as z-scores in heatmaps.

To reliably separate stage CV individuals by the program without a priori knowledge, we employed three strategies to identify distinguishing gene expression patterns embedded in the data (Fig. 1). The first strategy applied a dimensionality-reduction algorithm, the t-Distributed Stochastic Neighbor Embedding technique (t-SNE) to cluster samples agnostically by similarity in gene expression patterns (Fig. 1)^40,41^. The second strategy focused on the identification of differentially expressed genes (DEGs) followed by downstream correlation network analysis and examination of predicted gene function (strategy 2a)^32,42,43^. The third approach was focused on functional analysis of expression differences and comparison with expected physiological and transcriptional differences (strategies 2b, 3)^23,35,36,44–48^. This targeted strategy builds computational filters to generate sets of genes based on relevant biological processes and gene ontology (GO) terms that reliably separate samples by the developmental program. The goal of the analysis was to design filters that identified processes that were independent of or minimally affected by the environment (culture vs field) and/or time (early vs. late).

## Results and discussion

### Transcriptional phenotypes and analysis of differentially expressed genes

**Fig. 2:**
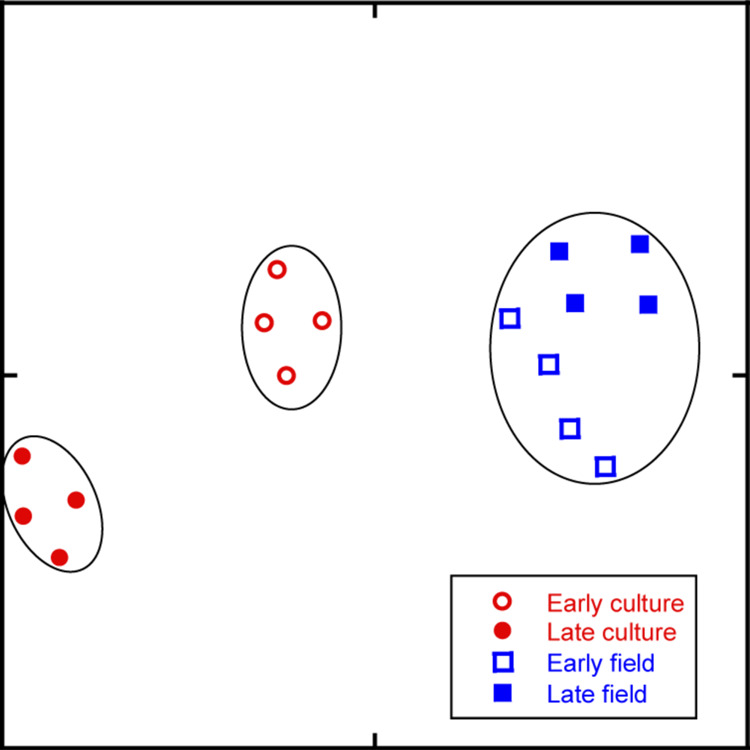
Dimensionality reduction and cluster identification of expression data from all genes using t-SNE and DBSCAN. Two-dimensional t-SNE plot of normalized and log-transformed expression profiles from mapped read data generated by Bowtie2 for the samples from the four groups (diapause program: EF, LF; reproductive program: EC, LC). The perplexity parameter was set to 5, and 50,000 iterations of the t-SNE algorithm were run. The DBSCAN algorithm was followed by the calculation of the Dunn index to determine the optimal grouping of points into clusters (enclosed in black ovals). Samples are coded by fill (open: early [E]; filled: late [L]) and by symbol and color (blue squares: field [F]; red circles: culture [C]).

Transcriptional phenotypic similarities among samples were assessed by applying the t-SNE algorithm to the *C. finmarchicus* expression data (see “Methods”). The t-SNE algorithm, widely used to distinguish among cell types within a single tissue, can identify homogeneous transcriptional phenotypic groups without a priori knowledge of “origin” or “treatment”^49,50^. It considers all transcripts simultaneously, grouping like phenotypes together, while excluding non-similar ones. The 16 samples aggregated into three separate clusters (Fig. 2). All field samples (diapause program), early (EF), and late (LF), belonged to the same transcriptional phenotype (i.e., in a single cluster), while the early (EC) and late (LC) culture samples (reproductive program) separated into distinct phenotypes.

Maturation during the CV stage for individuals on the reproductive program involves large changes in expression as reported previously^38^. The LC individuals were approaching the final molt, while the EC individuals were only about ¼ of the way through the ~2-week molt cycle (see “Methods”). The difference between early and late culture could not be attributed to “environmental” factors, since culture conditions remained constant during the experiment, nor could they be attributed to differences in the program since none of the cultured copepods entered diapause. In contrast, the field-collected individuals clustered together, despite the two-week separation between sampling points. The samples presumably derived from the same population in the fjord and represent different stages in progress within the diapause program. Asynchrony within the field population might have partially obscured any temporal changes in expression. However, because diapause involves the developmental arrest and a lengthening of life span, the similarity in expression patterns between early and late fields may well be intrinsic to CVs on the diapause program.

### Differential gene expression and functional analysis

A generalized linear model (GLM) identified over 11,000 DEGs among the four treatments (Table 1; strategy 2, Fig. 1). Consistent with the t-SNE results the smallest number of DEGs were found between the two sets of field samples, while the largest numbers of DEGs were between late culture CVs and those collected from the field (early and late field). The analysis also identified a large number of DEGs between early and late culture (6908), which is similar to the number reported previously for this comparison (“unique comps:” 7470) using a different reference transcriptome and short-read mapping program^38^.

**Table 1:** Statistical comparison of gene expression across four groups of stage CV *Calanus* that differed by source (field/diapause program vs. culture/reproductive program) and time point (early vs. late).

Statistical test	Comparison	DEGs	Upregulated	Downregulated
Generalized linear model		11,503		
Pairwise likelihood ratio test	EF vs LF	1739	982	757
	EF vs EC	7197	3926	3271
	EF vs LC	10,077	3090	6987
	LF vs EC	7675	3960	3715
	LF vs LC	9939	5056	4883
	EC vs LC	6908	3090	3818

Summary of the number of differentially expressed genes (DEGs). DEGs were identified using a generalized linear model followed by downstream pairwise likelihood ratio tests (significant for *p*-value ≤ 0.05; *p*-value adjusted with the Benjamini–Hochberg procedure to control for false discovery rate [FDR]). Field-collected: EF (early field), LF (late field). Culture: EC (early culture), LC (late culture).

**Fig. 3:**
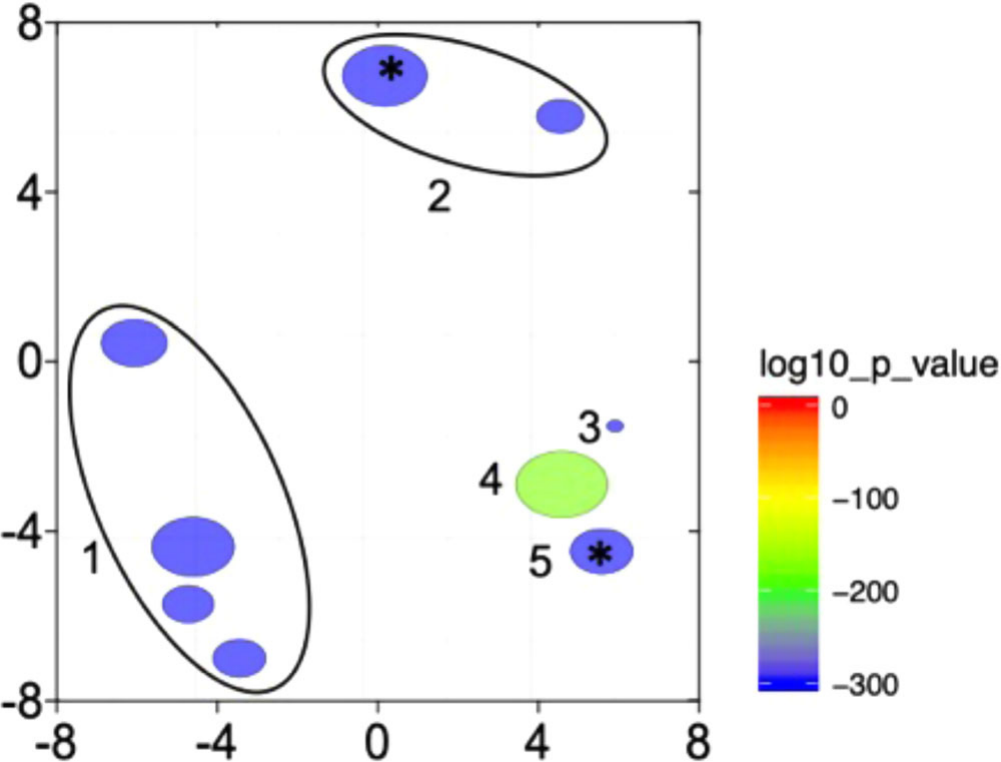
Biological processes represented among the differentially expressed genes (DEGs). ReviGO two-dimensional representation of all GO terms represented among the total number of annotated DEGs (*n* = 5509). The redundancy reduction filter was set to “small” (0.5). Each “bubble” represents a GO term; bubble color scales by the *p*-value determined by EdgeR (color scale, bottom right) and the bubble size indicates the frequency of the GO term in the underlying gene-ontology annotation database. Based on the Gene Ontology hierarchical organization, GO terms with the same GO parent have been circled (black line) and are indicated as a single number. GO term annotation: (1) Development/reproduction (‘developmental process involved in reproduction’ [GO:0003006], ‘reproduction’ [GO:0000003], ‘cellular developmental process’ [GO:0048869], ‘germ cell development’ [GO:0007281]). (2) Lipid metabolism (‘lipid metabolic process’ [GO:0006629], *‘long-chain fatty acid metabolic process’ [GO:0001676]); (3) ‘Response to stress’ [GO:0006950]; (4) ‘Signal transduction’ [GO:0007165]; (5) *‘RNA metabolic process’ [GO:0016070]. Asterisks (*) mark GO terms that were already represented among the DEGs but were significantly enriched ([GO:0001676] and [GO:0016070]).

A search of the reference transcriptome for functional annotations returned nearly half of the DEGs with gene ontology (GO) terms (*n* = 5509; strategy 2, Fig. 1). The ReviGO summarization grouped DEGs into nine GO terms (Fig. 3). The broad categories of ‘development’ (group 1) and ‘lipid metabolism’ (group 2) included four and two GO terms respectively. Development included GO terms associated with reproduction (e.g., ‘germ cell development’, ‘developmental process involved in reproduction’). Enrichment analysis identified two metabolic processes as over-represented among the DEGs (‘very long-chain fatty acid metabolic process’ and ‘RNA metabolic process’). These processes might well be expected to be over-represented between individuals on reproductive vs diapause programs.

**Fig. 4:**
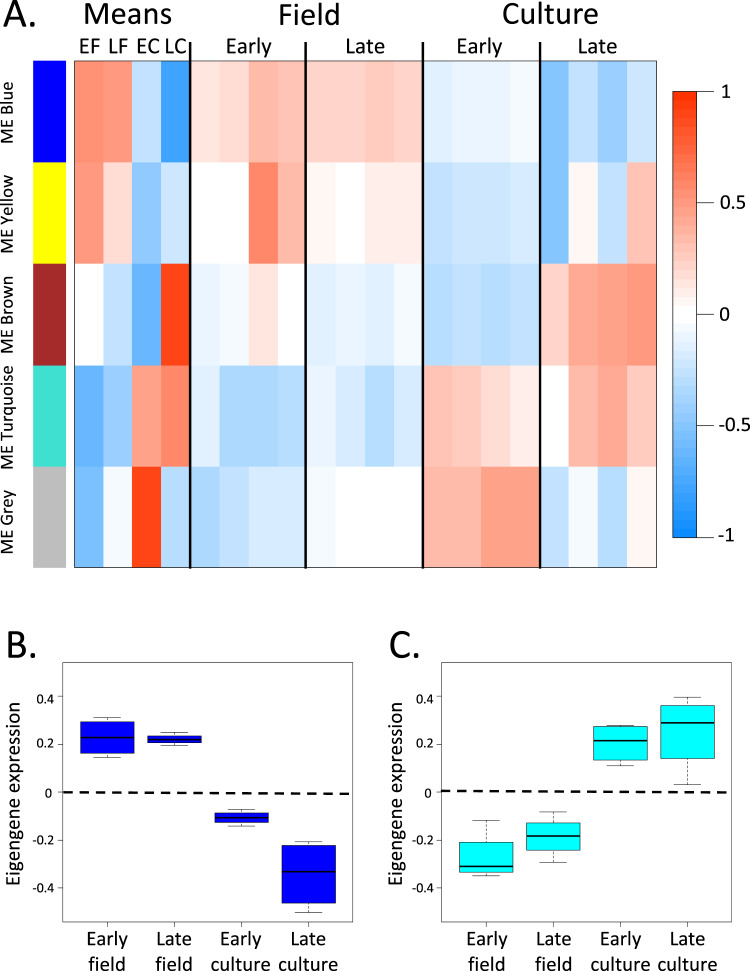
Correlation network analysis (WGCNA) of DEGs showing modules of genes with similar expression patterns. **A** WGCNA network significance correlation matrix. Heatmap of correlation of module eigengenes with sample traits (rows correspond to modules [labeled by color] and columns to groups or individual samples). The first four columns represent the correlation of module eigengenes with each group (diapause program: early field [EF], late field [LF]; reproductive program: early culture [EC], late culture [LC]). Columns on the right (16) are the correlations of the eigengene expression for each module with the individual samples. Direction and the strength of correlation are indicated by color with blue showing negative (downregulation) and red showing positive (upregulation) (color scale on right). Number of genes in the four major modules: blue (*n* = 3827), yellow (*n* = 745), brown (*n* = 1133), turquoise (*n* = 5689). A small number of DEGs were placed into the “unassigned” gray module (*n* = 109). **B** Box and whiskers plot of the blue module eigengene expression for each group (*n* = 4). **C** Box and whiskers plot of the turquoise module eigengene values for each group (*n* = 4). The box displays the median and interquartile range, while the whiskers give the minimum and maximum values for each group.

Differences between samples were further analyzed using correlation network analysis (WGCNA) to group DEGs into modules with highly correlated expression patterns (strategy 2a, Fig. 1). WGCNA identified four modules using the 11 K DEGs. The largest numbers of DEGs were assigned to two modules (blue > 3500; turquoise > 5000) (Fig. 4A). Expression patterns of these two modules differentiated between field and culture samples, as shown in the box and whiskers plots of module eigengene expression (Fig. 4B, C, see “Methods”). DEGs in the blue module were positively correlated with CVs on the diapause program (warm colors in field, cool colors in culture), while the DEGs in the turquoise module had the opposite expression pattern (Fig. 4A). Enrichment analysis of the GO terms represented among the DEGs identified a single over-represented process in each module: ‘glycerophospholipid biosynthetic process’ (GO:0046474) in the blue module and ‘positive regulation of RNA metabolic process’ (GO:0051254) in the turquoise module. ‘Glycerophospholipid biosynthetic process’ is associated with the formation of glycerophospholipids, which are constituents of membranes and lipoproteins. It is a ‘child term’ of ‘lipid metabolic process’ (GO:0006629; bubble 2, Fig. 3). ‘Positive regulation of RNA metabolic process’ is a child term of ‘RNA metabolic process’ (GO:0016070), which was identified as an enriched process in the overall analysis (bubble 5, Fig. 3). In summary, this analysis identified only two key biological processes that drive gene expression differences between culture/reproduction and field/diapause programs.

### Transcriptional analysis of expected physiological differences

**Fig. 5:**
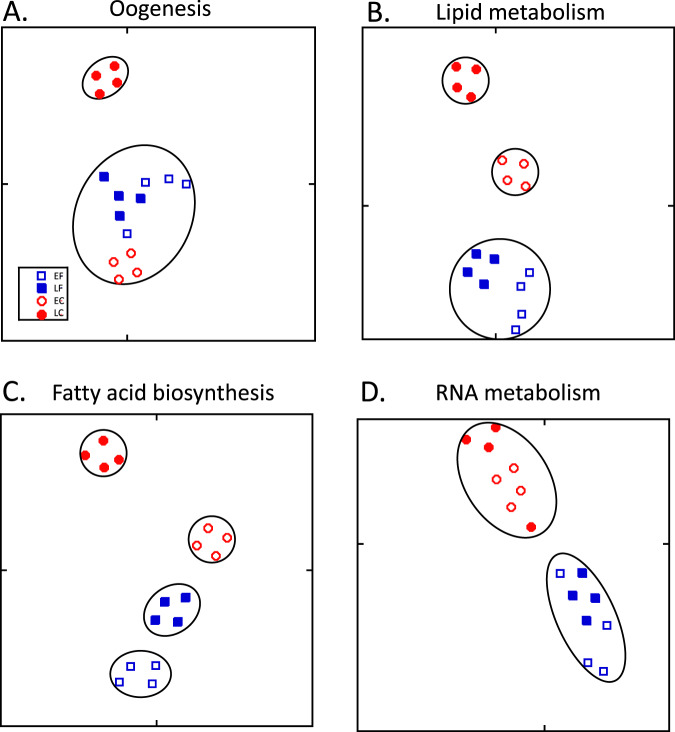
t-SNE plots for subsets of transcripts filtered according to membership in different gene ontology (GO) terms and their child terms. Circular profiles enclose clusters as determined by DBSCAN algorithm. **A** ‘Oogenesis’ filter [GO:0048477]; **B** ‘Lipid metabolic process’ filter [GO:0006629]; **C** ‘Fatty acid biosynthesis’ filter [GO:0006633]; **D** ‘RNA metabolic process’ filter [GO:016070]. Only the ‘RNA metabolic process’ filter divided the samples into separate field and culture transcriptional phenotypes. Symbol coding: field samples [F]: squares; culture samples [C]: circles; early samples [E]: open symbols; late samples [L]: closed symbols. All panels: perplexity = 5; number of iterations = 50,000 identified using DBSCAN with MinPts = 3 and the Eps value that maximized the Dunn index.

The goal of the third strategy was to analyze differences in expression by employing a priori knowledge on differences in developmental, metabolic and regulatory processes described in insects and expected in copepods^23,36,45,46,51^. Diapause preparation includes metabolic changes that lead to fat accumulation in arthropods and the build-up of wax ester stores in *C. finmarchicus* and other calanid copepods^20,23,30,47,52–55^. In contrast, maturing females require energetic resources for provisioning oocytes^17,56^. The differential gene expression analysis presented above broadly supports this, but has provided few details. In combination with heatmaps of the DEGs, we used gene ontology (GO) filters to establish transcriptional phenotypes associated with all genes annotated to specific processes in the reference transcriptome independent of whether they were differentially expressed (strategy 3, Figs. 1, 5).

**Fig. 6:**
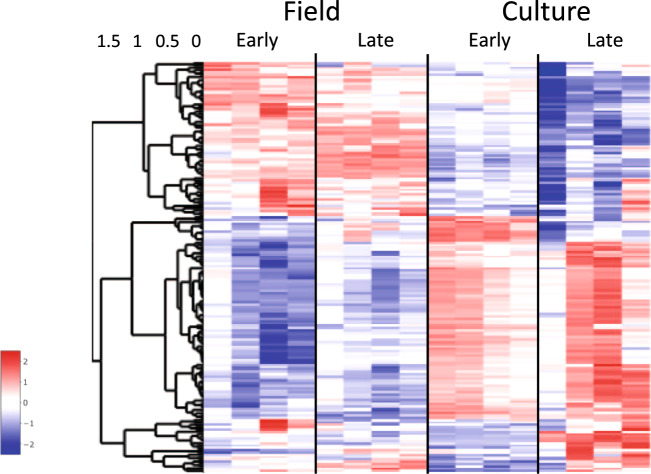
Expression heatmap showing z-scores of DEGs involved in oogenesis. Differentially expressed genes between field/diapause program and culture/reproductive program and early and late CV copepodids annotated with the ‘oogenesis’ [GO:0048477] GO term and its child terms (*n* = 178). Color-coding for each gene indicates the magnitude of expression as z-scores of each individual sample. Relative expression of each sample is given in a separate column (ordered by group) as labeled at the top. Genes (rows) were ordered by hierarchical clustering.

Gonad development and early oogenesis occur during stage CV in individuals on the reproductive^57^, but not the diapause program, a difference that was confirmed by microscopic examination of cultured and field-collected individuals done concurrently with the transcriptomics^23^. In the reference transcriptome, 584 genes were annotated to oogenesis (GO:0048477, and its child terms). Dimensionality reduction by t-SNE of relative expression of these genes separated the 16 samples into two clusters (Fig. 5A). The late culture individuals, which were approaching maturity, aggregated into a distinct cluster. However, there was no substantial separation of the 12 remaining samples, which were widely distributed within a single cluster. A heatmap of relative expression of the 178 DEGs annotated with the oogenesis GO term is consistent with the t-SNE result: somewhat more than half of the oogenesis genes were upregulated and the rest downregulated in late culture CV samples when compared with all other samples (Fig. 6). For the remaining 12 samples, even if more variable, the expression pattern of several genes separated the field samples from the early culture samples. Thus, a general oogenesis filter may prove useful in the identification of CVs approaching the final molt, but it may be less successful in separating recently molted CVs on the reproductive program from those on the diapause program.

Genes involved in lipid metabolism are expected to be differentially expressed between reproductive- and diapause-program CV individuals given fat accumulation during diapause preparation^23^. Processes linked to lipid metabolism were found to be enriched among all DEGs, and a child term was enriched in the blue (diapause-correlated) module of the WGCNA analysis. To pursue this further, two lipid metabolism filters were applied to the whole transcriptome: ‘lipid metabolic process’ (GO:0006629 and its child terms) with 717 genes and ‘fatty acid biosynthetic process’ (GO:0006633 and its child terms) with 70 genes. A t-SNE analysis that included all genes annotated to the first of these separated the 16 samples into three clusters (Fig. 5B) that were qualitatively similar to the t-SNE analysis that included all genes (Fig. 2). The culture samples segregated into an early and a late group suggesting that maturation during the CV stage includes regulation of lipid metabolism.

**Fig. 7:**
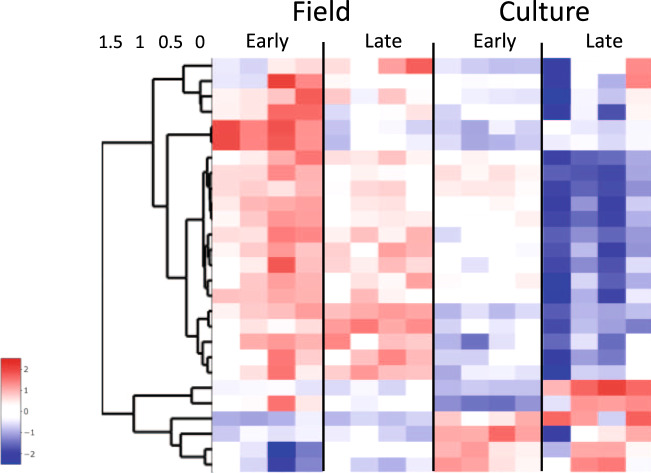
Expression heatmap showing z-scores of DEGs involved in fatty acid biosynthesis. Differentially expressed genes between field/diapause program and culture/reproductive program and early and late CV copepodids annotated with the GO term ‘fatty acid biosynthetic process’[GO:0006633] or its child terms (*n* = 23). Color-coding for each gene indicates the magnitude of expression as z-scores of each individual sample. Relative expression of each sample is given in a separate column (ordered by group) as labeled at the top. Genes (rows) were ordered by hierarchical clustering.

The more specific filter of ‘fatty acid biosynthesis’ separated the samples into four clusters, with the early and late field samples placed into distinct transcriptional phenotypes (Fig. 5C). Such a pattern could be explained by the regulation of fatty acid metabolism along the CV’s progression towards diapause in the field, and/or it could reflect responses to environmental differences between the two sampling times. Thus, a limitation of a GO filter associated with lipid metabolism is that expression differences may occur in response to environmental factors such as food quantity and quality, as reported in another diapausing calanid, *Neocalanus flemingeri*^40,42^. Nevertheless, the 23 DEGs annotated to ‘fatty acid biosynthesis process’ (GO:0006633) show a general upregulation of genes associated with lipid synthesis in field-collected individuals in comparison with cultured individuals (Fig. 7). However, the expression pattern was quite variable among individual samples. Consistent with diapause preparation in field CVs, we observed the upregulation of enzymes involved in wax ester biosynthesis (two *diacylglycerol O-acyltransferases 1* and two *fatty acyl-CoA reductases*), a process directly related to lipid accumulation.

Downregulation of genes involved in RNA and DNA metabolism during diapause has been demonstrated in insects, copepods, and other arthropods^32,36,58^. While the environmental triggers of diapause in calanid copepods remain unknown, in insects the developmental program can be pre-set by varying day length. This allowed Poelchau and colleagues to compare gene expression in non-diapause (“ND”) with diapause-bound (“D”) individuals in the mosquito *Aedes albopictus* during embryogenesis^35^. Downregulation of genes involved in metabolism, energy production, and protein synthesis, including a child term of ‘RNA metabolic process’ was already apparent during pre-diapause. A similar pattern was observed in *C. finmarchicus*. Genes involved in ‘RNA metabolic process’ were downregulated in field CV individuals and this process was enriched among the DEGs (Figs. 3, 4, see turquoise module).

**Fig. 8:**
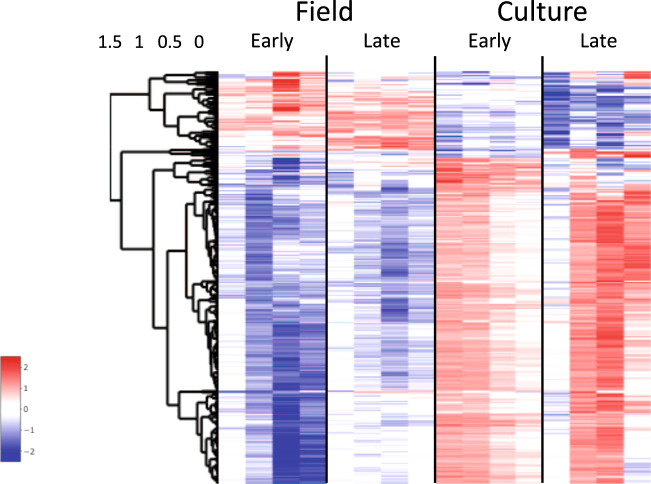
Expression heatmap showing z-scores of DEGs involved in RNA metabolism. Differentially expressed genes between field/diapause program and culture/reproductive program and early and late CV copepodids annotated with the GO term ‘RNA metabolic process’ [GO: 0016070] or its child terms (*n* = 335). Color-coding for each gene indicates the magnitude of expression as z-scores of each individual sample. Relative expression of each sample is given in a separate column (ordered by group) as labeled at the top. Genes (rows) were ordered by hierarchical clustering.

Application of a general filter for ‘RNA metabolic process’ (GO:0016070 and child terms, *n* = 1064) followed by t-SNE separated the 16 samples into two clusters consisting of either culture or field samples (Fig. 5D). This filter did not show differences in gene expression associated with maturation in CVs on the reproductive program (i.e., clustering both EC and LC together). The pattern in the heatmap of 335 DEGS is consistent with the t-SNE analysis and clearly separated the field from the culture samples, in spite of individual variability among replicate samples (Fig. 8). Most of these DEGs showed low expression in diapause-bound (field) individuals and substantially higher expression in culture individuals. Among the DEGs are several genes encoding proteins involved in RNA processing such as *pre-mRNA splicing factors*, *spliceosomes,* and *mRNA decay activators* (Fig. 8). This signal was more pronounced and pervasive in *C. finmarchicus* than in the mosquito^35^. While it is possible that environmental factors contributed to this separation of culture and field individuals, neither ‘RNA metabolic process’ nor any of its child terms were identified as enriched among the differentially expressed genes reported in diapause-bound *N. flemingeri* collected from locations with order of magnitude differences in food resources^42^.

**Fig. 9:**
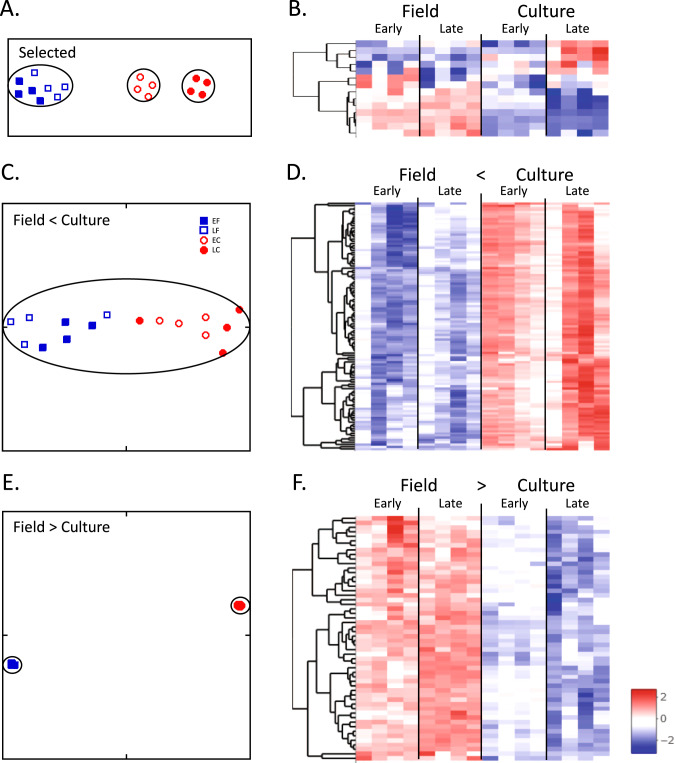
“Designer” filters with high-responding genes to separate field from culture transcriptional phenotypes. **A**, **B** Filter selecting genes based on evidence for involvement in diapause preparation, as described in the text. **C**–**F** Transcripts were selected from DEGs annotated with the GO terms ‘oogenesis’ (GO:0048477), ‘fatty-acid biosynthesis’ (GO:0006633) and ‘RNA metabolic process’ (GO:0016070). **C**–**D** Filter selecting transcripts having z-scores in all culture samples higher than any in the field samples. **E**–**F** Filter selecting transcripts having z-scores in all field samples higher than any in the culture samples. **A**, **C**, **E** t-SNE plots, perplexity = 5; max iterations 50,000; clusters identified by DBSCAN encircled. Key in (**C**) applies to all t-SNE plots: EF early field, LF late field, EC early culture, LC late culture; **B**, **D**, **F** ordered heatmaps.

Another approach to identify calanids on the diapause program has been to explore potential biomarker genes by selecting a set of genes a priori based on comparisons between presumably active or dormant field-collected individuals. Such comparisons exist for *C. finmarchicus* and *Calanus sinicus* with samples collected from different depths and profiling relative gene expression using a variety of molecular methods^59–61^. Differentially expressed genes from these studies were then cross-referenced to genes regulated just prior to diapause in insects, *Artemia* and/or *Caenorhabditis elegans*^35,62–65^. Using this approach, we identified 14 potential candidates for biomarker genes (Fig. 9A, B, Supplementary Table S1). These genes did not include any annotated to GO terms used in the previous filters. Based on our analysis, seven genes were differentially expressed (three *serpins* [out of 4], two *nitric oxide synthases* [out of eight], one *phosphoenolpyruvate carboxylase kinase* [out of 1], one *RAS-related protein Rab-10* [out of 1]), and relative expression of these genes differed between field and culture as shown in the heatmap (Fig. 9B). However, a t-SNE analysis of the relative expression of these 14 genes failed to separate the samples into cohesive field and culture clusters, but rather generated three clusters, similar to the pattern generated in the initial analysis that included all genes (Fig. 9A).

### Workflow to separate CVs by program using RNA-Seq of individuals

While we focused on a set of 16 pooled RNA-Seq samples from four known treatment groups, the goal was to develop a protocol to determine which and how many CVs are on the diapause program in a natural population. Gene expression profiles generated for individual CVs collected from the environment could be assessed for the developmental program by producing expression profiles for the ca. 1000 genes annotated to ‘RNA metabolic process’ in the reference transcriptomes and applying t-SNE. We hypothesize that applying t-SNE to these profiles will separate individuals into two clusters based on developmental program, which can then be confirmed using differential gene expression. Individuals can be separated by cluster membership and tested for expected gene expression differences between the diapause and reproductive programs.

Another approach is a search for robust indicator genes. Ecological studies require testing large numbers of individuals across time and space, which calls for protocols capable of high-throughput of samples based on RT-qPCR or nCounter (NanoString®) technologies^66^. These technologies need a smaller set of indicator genes with a robust signal-to-noise ratio (Fig. 9). We searched for a set of genes with consistent and large differences in expression between culture and field samples among the DEGs annotated to the three GO terms that we tested for differentiating between programs (oogenesis, GO:0048477, *n* = 178; fatty-acid biosynthesis, GO:0006633 *n* = 23; and RNA metabolic process, GO: 0016070, *n* = 335). A transcript was included when all of its expression z-scores in culture samples were either above or below all of its values among the field samples (i.e., no overlap in relative expression). This selection method is clearly shown in the respective heatmaps for the two filters with all of one color for the field and the opposite color for the culture samples (Fig. 9D, F). Genes that were upregulated in culture (i.e., reproductive program) compared with field included 111 such transcripts from oogenesis (*n* = 32) and RNA metabolic process (*n* = 79), but none from fatty acid biosynthesis passed this filter (Fig. 9C, D). Relative expression of these genes was highly variable and did not separate the CVs by the developmental program as shown by the single cluster in the t-SNE plot (Fig. 9C).

In contrast, a filter comprising genes that were more highly expressed in the field (i.e., diapause program) than culture samples, produced two tight and distinct clusters in t-SNE (Fig. 9E). The 54 transcripts in this filter included representatives from all three GO terms: oogenesis (*n* = 19), RNA metabolic process (*n* = 28), and fatty acid biosynthesis (*n* = 7) as shown in the heatmap (Fig. 9F; Supplementary Table S2). While it is premature to speculate on their specific functions with respect to the diapause program in *C. finmarchicus*, these genes are good candidates for further investigation. Two transcripts on this list, one *diacylglycerol O-acyltransferase 1* and one *fatty acyl-CoA reductase* are predicted to be involved in wax ester biosynthesis, while another *AMP-activated protein kinase* (*AMPK*) *gamma 1* is involved in the regulation of cellular energy metabolism.

## Conclusions

An existing RNA-Seq dataset was analyzed to develop a workflow for environmental transcriptomics that can classify pre-adult CV *C. finmarchicus* individuals by developmental program. Through a combination of statistical and functional analyses, we propose two workflows. The first relies on a global gene expression analysis (RNA-Seq) and involves applying a gene ontology filter (RNA metabolic process) followed by t-SNE clustering to separate samples into groups for statistical comparison. The second workflow employs an indicator strategy for high-throughput gene expression technologies. A designer filter identified 54 genes that were consistently upregulated in individuals on the diapause program compared with those on the reproductive program. The t-SNE analysis of the relative expression of these genes separated the samples into two distinct transcriptional phenotypes based on the developmental program. While these workflows need further testing in natural populations, they may be broadly applicable to *C. finmarchicus* and other diapausing calanid copepods. These molecular approaches can be used to assess reproductive strategies within an environmental context. Furthermore, the specific genes and pathways identified in this analysis may be good candidates for elucidating the physiological processes that differentiate the two developmental programs, including determining when the decision to diapause is made in copepods.

## Methods

### *Calanus finmarchicus* reference transcriptome

The study used an existing Gulf of Maine *Calanus finmarchicus* transcriptome for mapping the short sequence reads (NCBI BioProject PRJNA236528)^67^. Briefly, this reference was assembled from 100 bp short-sequence reads from six developmental stages and had been annotated against the SwissProt protein database (www.uniprot.org). Annotation identified 28,616 transcripts with significant similarity to known proteins (*E*-value cutoff = 10^−3^) and 10,334 transcripts with significant GO annotations (E-value cut-off = 10^−6^; http://geneontology.org/)^67–69^. The reference with 96 K transcripts had no contamination from other *Calanus* species and was characterized by very low ambiguous mapping (<1% ‘mapped more than once’ by Bowtie2)^68^.

### RNA-Seq data description, retrieval, and pre-processing

Short-read sequences for 16 samples were downloaded from the short-sequence read archive (SRA) in the National Center for Biotechnology Information (NCBI) database (Table S1, Supplementary Note; Illumina HiSeq2000, 50 bp, paired-end with ≥30 M spots per sample, (BioProject: PRJNA 231164)^38^. For each sample, RNA had been extracted from pools of stage CV individuals (5–7)^38^. The dataset included four replicate samples for each of the two time-points in both the laboratory-cultured population and the field-collected wild population^23,38^. The experimental design and number of replicates provided the necessary statistical power for this analysis, which focused on distinguishing between two developmental programs. Additional details on the experiments can be found in previous studies^23,38^ and in the biosample descriptions in the NCBI database. Previous analysis of the data focused on characterizing transcriptional changes associated with maturation in stage CV copepodids on the reproductive program^38^. In a second study, differences in the developmental program were sought by analyzing pathways associated with lipid metabolism for temporal changes in gene expression of biomarkers in culture and field CVs using RT-qPCR. While differences in relative expression were noted, this analysis was not detailed enough to discriminate between “within stage maturation” and developmental program^23^. Neither study included an analysis of the high-throughput sequencing of the field samples, which is the central approach used in the current study.

Briefly, the laboratory-cultured samples consisted of recently molted (≤24 h) stage CV copepodids that had been isolated and incubated separately until harvested at three (early culture, “EC”) and 10 days (late culture, “LC”) post-molt. The time points represented early and late stages in the molt cycle, which under the experimental conditions had a median duration of 13.5 days^38^. During the incubation, copepods were maintained on the standard culture diet^38,39^. Microscopic examination of other individuals from each experimental set of CVs confirmed the presence of early development of gonads at both three and 10 days post-molt. At three days post-molt, all individuals were in the pre-apolysis jaw phase, and by days 9 through 11, 45% had matured into post-apolysis jaw phases consistent with progression toward the terminal molt.

The diapause-program copepodids had been collected from the field at Trollet Station in Trondheimsfjord (63°29′N, 10°18′E) with a zooplankton net towed vertically from 50 to 0 m on 28 May 2013 (early field, “EF”) and 14 days later on 10 June 2013 (late field, “LF)^23^. Microscopic examination of CVs revealed that, consistent with pre-diapause, all had undifferentiated gonads and were in the pre-apolysis jaw phase^23^. The juvenile copepods were not sorted according to sex, and presumptive males and females were included in laboratory and field samples. Although the field samples were originally thought to contain only *C. finmarchicus* CVs, recent studies reported that *C. glacialis* and *C. helgolandicus* can co-occur with *C. finmarchicus* in the region including Trondsheimfjord^70–72^. The three congeners are morphologically very similar and can only be identified reliably to species using genetic tools (Choquet et al.^71^). We confirmed the presence of the congeners in the field samples using a molecular approach (see below, Supplementary Note).

In Trondsheimfjord, *C. glacialis* and *C. helgolandicus* are on the same diapause-bound program as is *C. finmarchicus*^73^, and thus are not expected to diverge greatly in their transcriptional phenotypes. Nevertheless, we examined the possibility of bias impacting the analysis due to species composition differences between field and culture samples. We concluded that there was no significant bias, and the multi-step analyses that led us to this conclusion are described in detail in the Supplementary Note.

Briefly, we assessed the species composition of each sample by using species differences in the mtCOI sequences^74^ and quantifying reads mapping to each sequence. Significant contamination was limited to the field samples. Congener composition of most samples was below 32%, which combined with an estimated 30% cross-mapping efficiency to congeneric references^75^, indicated a modest 11% estimate for mean cross-mapping levels (Table S1, Supplementary Note). We then used publicly available congeneric read sets to identify the most cross-map-prone transcripts in our *C. finmarchicus* reference. About half of the transcripts susceptible to cross-mapping were among the transcripts with significant expression (>1 count per million reads [cpm]). This proportion was maintained in most of the analyses we performed in our more targeted transcript selections, indicating a uniform contribution from cross-mapped sources (Table S2, Supplementary Note). However, there was some enrichment of cross-mapped transcripts, so in our last test we compared the t-SNE analyses for each transcript set with a paired set that excluded all transcripts with cross-mapped reads (Fig. S1, Supplementary Note). The effects were minimal, and duplicated the transcriptional phenotype results when the conserved transcripts with some contamination from cross-mapped reads were included in the set. Thus, the main text refers to samples as *C. finmarchicus* samples, this being the dominant species present and the species used as the bioinformatic reference.

### Mapping of short reads and computation of relative gene expression

After quality filtering to remove sequences with a Phred score ≤20, short sequence reads from each sample were mapped against the *C. finmarchicus* reference transcriptome to generate gene expression profiles (Fig. 1) using Bowtie2 software (default settings; v.2.1.0)^76^ (Table S1, Supplementary Note). After the mapping step, RPKM (reads per kilobase of transcript length per million mapped reads) were calculated to normalize relative gene expression [i.e., for transcript i from sample j, RPKM(i,j) = reads(i,j)/[(length(i)/1000)*(mapped_reads(j)/1000000)]^77^. We next log_2_ transformed the relative expression data after adding a pseudocount of 1 to the RPKM value for each transcript (i.e., log_2_[RPKM+1]) (Fig. 1). These log-transformed relative expression data were used in all dimensionality-reduction analyses and to calculate z-scores for each transcript and sample. Z-scores were used in heatmaps for expression comparisons across samples.

### Dimensionality reduction and identification of transcriptional phenotypes

The dimensionality reduction method t-distributed Stochastic Neighbor Embedding (t-SNE) was used to visualize variation in gene expression across samples^41,78^ (Fig. 1, strategy 1). The t-SNE algorithm reduces the high dimensional gene expression profiles to a two-dimensional representation while seeking to conserve the local relationships among the samples. We have found t-SNE to be better for identifying copepod transcriptional phenotypes than other dimensionality-reduction methods such as principal component analysis (PCA)^40^. We applied t-SNE as implemented in the R package Rtsne (*Rtsne* URL: https://github.com/jkrijthe/Rtsne)^79^ to the log-transformed RPKM values for either the entire set of transcripts (*n* = 96,090), or for subsets of transcripts filtered for specific GO terms (see below; Fig. 1, strategy 3). After pre-testing, program parameters were set as follows: perplexity = 5, maximum number of iterations = 50,000 and the remaining parameters equal to their default values. In addition, the t-SNE algorithm was run multiple times to ensure that the output was representative (i.e., to ensure that the phenotypes so identified were robust)^40^. The results were plotted as a 2-D scatterplot in the t-SNE coordinates. To provide an objective method of identifying which samples formed clusters, the density-based clustering algorithm, DBSCAN (with *MinPts* = 3) was applied to the t-SNE results (coordinates of points)^40,80^. The clustering cut-off (*Eps* parameter) was chosen to maximize the Dunn index score^81^. Both the DBSCAN algorithm and the Dunn index were run in R (dbscan: https://CRAN.R-project.org/package=dbscan; clusterCrit: https://CRAN.R-project.org/package=clusterCrit)^40,82,83^.

### Differential gene expression and weighted gene correlation network analysis (WGCNA)

The “mapped reads” file generated by *Bowtie2* was used as the input to the BioConductor package EdgeR to identify differentially expressed genes (DEGs)^84^ (Fig. 1, strategy 2). Prior to the statistical analysis, transcripts with very low expression levels (those failing to have at least 1 cpm in 4 of the 16 samples) were removed leaving a total of 27,870 transcripts (out of the original 96,060 in the reference). As implemented by EdgeR, libraries were normalized using the TMM method (trimmed mean of M values). The negative binomial generalized linear model (GLM) identified DEGs across samples (glmFit function) with *p*-values adjusted for false discovery rate (FDR; Benjamini–Hochberg procedure). The GLM analysis was followed by pairwise comparisons using the downstream likelihood ratio test (glmLRT) to identify significant differences in gene expression between each treatment pair (*p*-value ≤ 0.05, corrected for FDR).

Patterns of differential gene expression among samples were explored using weighted gene correlation network analysis (WGCNA), a technique for finding modules of highly correlated genes across treatments^85,86^. Downstream analysis of modules or a representative of the gene expression profiles in each module, such as the “eigengene”, provides a network-based method for data reduction. The WGCNA analysis was performed on the log-transformed (log_2_[RPKM+1]) gene expression of all DEGs (11 K, Fig. 1, strategy 2). The analysis used an unsigned, weighted network with a soft threshold power of 14 and minimum module size (minModuleSize) set to 100. Modules were determined by applying the automatic block-wise module detection function of the WGCNA package. The module eigengene, defined as the first principal component of the module gene expression matrix, gives a weighted average of the module expression profiles and was used to investigate the relationship between modules and biologically interesting sample traits. Pearson correlations between module eigengenes and membership in a specific experimental group were computed. A heatmap was generated to visualize these correlations by experimental group and for the individual samples to allow comparison of expression patterns across replicates. We used boxplots to display the descriptive statistics (median, first (25%) quartile, third (75%) quartile, minimum and maximum) of module eigengene expression for each experimental group (EF, LF, EC, and LC). Annotated genes assigned into WGCNA modules were tested for enriched GO terms (see below).

### Functional analysis and filtering of genes using gene ontology

Functional analysis of the DEGs was based on the *C. finmarchicus* transcriptome. Briefly, DEGs were cross-referenced with the annotated transcriptome and nearly half were found to have GO term annotations. ReviGO software was used to summarize and visualize in two-dimensional space the biological processes represented among the DEGs^87^. The list of GO-annotated DEGs (all) and their *p*-values were summarized using a very stringent filter (similarity setting to “small” = 0.5), which substantially reduced the redundancy intrinsic to the Gene Ontology hierarchy.

Enrichment analysis was performed using TopGO software^88^ on DEGs with GO annotations. As implemented by TopGO, a Fisher exact test with a Benjamini–Hochberg correction (p-values ≤ 0.05 [v. 2.88.0, set to the default algorithm “weight01”]) was used to compare the DEGs identified for each sample pair (*n* = 6) against all transcripts with GO terms in the reference transcriptome^67^.

Based on the enrichment results (strategy 2, Fig. 1) and pre-determined functional hypotheses (strategy 3), several GO filters were applied to workflow strategies 2 and 3 (Fig. 1). Specifically, the AmigGO software GO Online SQL Environment (GOOSE)(October, 2019: http://amigo2.berkeleybop.org/goose/cgi-bin/goose) was used to search descendants of target GO terms to obtain all transcripts annotated to a specific process. For this, the LEAD SQLwiki on the AmiGO Labs prototype page, using the example called “find descendants of the node ‘nucleus’ was edited to replace ‘nucleus’ with the specific GO term to be used for the filter. The annotated reference transcriptome was then used to retrieve all transcripts within each functional category defined by specific GO terms and their child terms. In addition, GO lists were searched for DEGs, and heatmaps were generated using z-scores (see above) and the software package heatmaply in R, which clusters genes by expression similarity (heatmaply: https://github.com/talgalili/heatmaply/)^89^.

### Reporting summary

Further information on research design is available in the Nature Research Reporting Summary linked to this article.

## Supplementary information

Supplementary Information

Reporting Summary

## Data Availability

The RNA-Seq data analyzed in this study are available on the National Center for Biotechnology Information (NCBI) database under the BioProject PRJNA 231164. The files generated in this study with relative expression per contig (counts, RPKM, log_2_[RPKM+1] and z-scores) are available in DryAd^90^.

## References

[1] Record NR (2018). Copepod diapause and the biogeography of the marine lipidscape. J. Biogeogr..

[2] Conover RJ, Corner EDS (1968). Respiration and nitrogen excretion by some marine zooplankton in relation to their life cycles. J. Mar. Biol. Assoc. UK.

[3] Kattner G (2007). Perspectives on marine zooplankton lipids. Can. J. Fish. Aquat. Sci..

[4] Beaugrand G, Brander KM, Lindley JA, Souissi S, Reid PC (2003). Plankton effect on cod recruitment in the North Sea. Nature.

[5] Coyle K (2011). Climate change in the southeastern Bering Sea: impacts on pollock stocks and implications for the oscillating control hypothesis. Fish. Oceanogr..

[6] Liu H, Bi H, Peterson WT (2015). Large-scale forcing of environmental conditions on subarctic copepods in the northern California Current system. Prog. Oceanogr..

[7] Peterson WT (2017). The pelagic ecosystem in the Northern California Current off Oregon during the 2014–2016 warm anomalies within the context of the past 20 years. J. Geophys. Res. Oceans.

[8] Bi H, Peterson WT, Lamb J, Casillas E (2011). Copepods and salmon: characterizing the spatial distribution of juvenile salmon along the Washington and Oregon coast, USA. Fish. Oceanogr..

[9] Kirby RR, Beaugrand G (2009). Trophic amplification of climate warming. Proc. R. Soc. B.

[10] Hirche H-J (1987). Temperature and plankton II. Effect on respiration and swimming activity in copepods from the Greenland Sea. Mar. Biol..

[11] Mahara N, Pakhomov EA, Jackson JM, Hunt BP (2019). Seasonal zooplankton development in a temperate semi-enclosed basin: two years with different spring bloom timing. J. Plankton Res..

[12] Hooff RC, Peterson WT (2006). Copepod biodiversity as an indicator of changes in ocean and climate conditions of the northern California current ecosystem. Limnol. Oceanogr..

[13] Keister JE, Di Lorenzo E, Morgan C, Combes V, Peterson W (2011). Zooplankton species composition is linked to ocean transport in the Northern California Current. Glob. Change Biol..

[14] Johnson CL (2008). Characteristics of *Calanus finmarchicus* dormancy patterns in the Northwest Atlantic. ICES J. Mar. Sci..

[15] Ji RB, Edwards M, Mackas DL, Runge JA, Thomas AC (2010). Marine plankton phenology and life history in a changing climate: current research and future directions. J. Plankton Res..

[16] Weydmann A, Walczowski W, Carstensen J, Kwaśniewski S (2018). Warming of Subarctic waters accelerates development of a key marine zooplankton *Calanus finmarchicus*. Glob. Change Biol..

[17] Niehoff B, Madsen S, Hansen B, Nielsen T (2002). Reproductive cycles of three dominant *Calanus* species in Disko Bay, West Greenland. Mar. Biol..

[18] Meise CJ, O’Reilly JE (1996). Spatial and seasonal patterns in abundance and age-composition of *Calanus finmarchicus* in the Gulf of Maine and on Georges Bank: 1977–1987. Deep-Sea Res. II.

[19] Fiksen Ø (2000). The adaptive timing of diapause–a search for evolutionarily robust strategies in *Calanus finmarchicus*. ICES J. Mar. Sci..

[20] Miller CB, Crain JA, Morgan CA (2000). Oil storage variability in *Calanus finmarchicus*. ICES J. Mar. Sci..

[21] Miller CB, Cowles TJ, Wiebe PH, Copley NJ, Grigg H (1991). Phenology in *Calanus finmarchicus* - Hypotheses about control mechanisms. Mar. Ecol. Prog. Ser..

[22] Speirs DC (2006). Ocean-scale modelling of the distribution, abundance, and seasonal dynamics of the copepod *Calanus finmarchicus*. Mar. Ecol. Prog. Ser..

[23] Tarrant AM (2016). Transcriptional profiling of metabolic transitions during development and diapause preparation in the copepod *Calanus finmarchicus*. Integr. Comp. Biol..

[24] Baumgartner MF, Tarrant AM (2017). The physiology and ecology of diapause in marine copepods. Annu. Rev. Mar. Sci..

[25] Wilson RJ, Banas NS, Heath MR, Speirs DC (2016). Projected impacts of 21st century climate change on diapause in *Calanus finmarchicus*. Glob. Change Biol..

[26] Jónasdóttir SH, Visser AW, Richardson K, Heath MR (2015). Seasonal copepod lipid pump promotes carbon sequestration in the deep North Atlantic. Proc. Natl Acad. Sci. USA.

[27] Jónasdóttir SH, Wilson RJ, Gislason A, Heath MR (2019). Lipid content in overwintering *Calanus finmarchicus* across the Subpolar Eastern North Atlantic Ocean. Limnol. Oceanogr..

[28] Varpe Ø (2012). Fitness and phenology: annual routines and zooplankton adaptations to seasonal cycles. J. Plankton Res..

[29] Denlinger, D. L., Yocum, G. D. & Rinehart, J. P. in *Insect Endocrinology* (ed Gilbert, L. I.) 430–463 (Academic Press, 2012).

[30] Hirche HJ (1996). Diapause in the marine copepod, *Calanus finmarchicus* - a review. Ophelia.

[31] Häfker NS (2018). *Calanus finmarchicus* seasonal cycle and diapause in relation to gene expression, physiology, and endogenous clocks. Limnol. Oceanogr..

[32] Roncalli V (2018). Physiological characterization of the emergence from diapause: a transcriptomics approach. Sci. Rep..

[33] Roncalli V, Cieslak MC, Hopcroft RR, Lenz PH (2020). Capital breeding in a diapausing copepod: a transcriptomics analysis. Front. Mar. Sci..

[34] MacRae TH (2010). Gene expression, metabolic regulation and stress tolerance during diapause. Cell. Mol. Life Sci..

[35] Poelchau, M. F., Reynolds, J. A., Elsik, C. G., Denlinger, D. L. & Armbruster, P. A. Deep sequencing reveals complex mechanisms of diapause preparation in the invasive mosquito, *Aedes albopictus*. *Proc. R. Soc. B* **280** (2013). 10.1098/rspb.2013.0143 PMC3619507 23516243

[36] Ragland GJ, Keep E (2017). Comparative transcriptomics support evolutionary convergence of diapause responses across Insecta. Physiol. Entomol..

[37] Koštál V (2006). Eco-physiological phases of insect diapause. J. Insect Physiol..

[38] Tarrant AM (2014). Transcriptional profiling of reproductive development, lipid storage and molting throughout the last juvenile stage of the marine copepod *Calanus finmarchicus*. Front. Zool..

[39] Jensen LK (2006). A multi-generation *Calanus finmarchicus* culturing system for use in long-term oil exposure experiments. J. Exp. Mar. Biol. Ecol..

[40] Cieslak MC, Castelfranco AM, Roncalli V, Lenz PH, Hartline DK (2020). t-Distributed Stochastic Neighbor Embedding (t-SNE): a tool for eco-physiological transcriptomic analysis. Mar. Genomics.

[41] van der Maaten L, Hinton G (2008). Visualizing data using t-SNE. J. Mach. Learn. Res..

[42] Roncalli V, Cieslak MC, Germano M, Hopcroft RR, Lenz PH (2019). Regional heterogeneity impacts gene expression in the sub-arctic zooplankter *Neocalanus flemingeri* in the northern Gulf of Alaska. Commun. Biol..

[43] Johnson KM, Wong JM, Hoshijima U, Sugano CS, Hofmann GE (2019). Seasonal transcriptomes of the Antarctic pteropod *Limacina helicina antarctica*. Mar. Env. Res..

[44] Denlinger DL (2002). Regulation of diapause. Annu. Rev. Entomol..

[45] Denlinger DL, Armbruster PA (2014). Mosquito diapause. Annu. Rev. Entomol..

[46] Hahn DA, Denlinger DL (2011). Energetics of insect diapause. Annu. Rev. Entomol..

[47] Sim C, Denlinger DL (2009). Transcription profiling and regulation of fat metabolism genes in diapausing adults of the mosquito *Culex pipiens*. Physiol. Genomics.

[48] Sim, C. & Denlinger, D. L. Insulin signaling and the regulation of insect diapause. *Front. Physiol*. **4**, 189 (2013). 10.3389/fphys.2013.00189 PMC3717507 23885240

[49] Andrews TS, Hemberg M (2018). Identifying cell populations with scRNASeq. Mol. Asp. Med..

[50] Habib N (2016). Div-Seq: single-nucleus RNA-Seq reveals dynamics of rare adult newborn neurons. Science.

[51] Arrese EL, Soulages JL (2010). Insect fat body: energy, metabolism, and regulation. Annu. Rev. Entomol..

[52] Hahn DA, Denlinger DL (2007). Meeting the energetic demands of insect diapause: nutrient storage and utilization. J. Insect Physiol..

[53] Lee RF, Hagen W, Kattner G (2006). Lipid storage in marine zooplankton. Mar. Ecol. Prog. Ser..

[54] Kattner G, Hagen W (1995). Polar herbivorous copepods–different pathways in lipid biosynthesis. ICES J. Mar. Sci..

[55] Miller CB, Morgan CA, Prahl FG, Sparrow MA (1998). Storage lipids of the copepod *Calanus finmarchicus* from Georges Bank and the Gulf of Maine. Limnol. Oceanogr..

[56] Hirche HJ, Niehoff B (1996). Reproduction of the Arctic copepod *Calanus hyperboreus* in the Greenland Sea-field and laboratory observations. Pol. Biol..

[57] Niehoff B, Hirche H-J (1996). Oogenesis and gonad maturation in the copepod *Calanus finmarchicus* and the prediction of egg production from preserved samples. Pol. Biol..

[58] Koštál V, Štětina T, Poupardin R, Korbelová J, Bruce AW (2017). Conceptual framework of the eco-physiological phases of insect diapause development justified by transcriptomic profiling. Proc. Natl Acad. Sci. USA.

[59] Aruda AM, Baumgartner MF, Reitzel AM, Tarrant AM (2011). Heat shock protein expression during stress and diapause in the marine copepod *Calanus finmarchicus*. J. Insect Physiol..

[60] Unal E, Bucklin A, Lenz PH, Towle DW (2013). Gene expression of the marine copepod *Calanus finmarchicus*: responses to small-scale environmental variation in the Gulf of Maine (NW Atlantic Ocean). J. Exp. Mar. Biol. Ecol..

[61] Ning J, Wang MX, Li CL, Sun S (2013). Transcriptome sequencing and *de novo* analysis of the copepod *Calanus sinicus* using 454 GS FLX. PLoS ONE.

[62] Zhang Q, Lu Y-X, Xu W-H (2013). Proteomic and metabolomic profiles of larval hemolymph associated with diapause in the cotton bollworm, *Helicoverpa armigera*. BMC Genomics.

[63] Hansen, M. et al. A role for autophagy in the extension of lifespan by dietary restriction in *C. elegans*. *PLoS Genet.* **4**, e24 (2008). 10.1371/journal.pgen.0040024 PMC2242811 18282106

[64] Qiu Z, MacRae TH (2008). ArHsp21, a developmentally regulated small heat-shock protein synthesized in diapausing embryos of *Artemia franciscana*. Biochem. J..

[65] Lu M-X (2013). Diapause, signal and molecular characteristics of overwintering *Chilo suppressalis* (Insecta: Lepidoptera: Pyralidae). Sci. Rep..

[66] Forreryd A, Johansson H, Albrekt A-S, Lindstedt M (2014). Evaluation of high throughput gene expression platforms using a genomic biomarker signature for prediction of skin sensitization. BMC Genomics.

[67] Lenz PH (2014). *De novo* assembly of a transcriptome for *Calanus finmarchicus* (Crustacea, Copepoda)–the dominant zooplankter of the North Atlantic Ocean. PLoS ONE.

[68] Roncalli V, Cieslak MC, Lenz PH (2016). Transcriptomic responses of the calanoid copepod *Calanus finmarchicus* to the saxitoxin producing dinoflagellate *Alexandrium fundyense*. Sci. Rep..

[69] Roncalli, V., Cieslak, M. C. & Lenz, P. H. Data from: Transcriptomic responses of the calanoid copepod *Calanus finmarchicus* to the saxitoxin producing dinoflagellate *Alexandrium fundyense*. *Dryad, Dataset* (2016). 10.1038/srep25708 PMC4867593 27181871

[70] Choquet M (2017). Genetics redraws pelagic biogeography of *Calanus*. Biol. Lett..

[71] Choquet M (2018). Can morphology reliably distinguish between the copepods *Calanus finmarchicus* and *C. glacialis*, or is DNA the only way?. Limnol. Oceanogr.: Methods.

[72] Skottene E (2019). A crude awakening: effects of crude oil on lipid metabolism in calanoid copepods terminating diapause. Biol. Bull..

[73] Melle W, Skjoldal HR (1998). Reproduction and development of *Calanus finmarchicus*, *C. glacialis* and *C. hyperboreus* in the Barents Sea. Mar. Ecol. Prog. Ser..

[74] Weydmann A (2017). Mitochondrial genomes of the key zooplankton copepods Arctic Calanus glacialis and North Atlantic Calanus finmarchicus with the longest crustacean non-coding regions. Sci. Rep..

[75] Lenz PH, Lieberman B, Cieslak MC, Roncalli V, Hartline DK (2021). Transcriptomics and metatranscriptomics in zooplankton: wave of the future?. J. Plankton Res..

[76] Langmead, B., Trapnell, C., Pop, M. & Salzberg, S. L. Ultrafast and memory-efficient alignment of short DNA sequences to the human genome. *Genome Biol*. 10.1186/Gb-2009-10-3-R25 (2009). 10.1186/gb-2009-10-3-r25 PMC2690996 19261174

[77] Mortazavi A, Williams BA, McCue K, Schaeffer L, Wold B (2008). Mapping and quantifying mammalian transcriptomes by RNA-Seq. Nat. Methods.

[78] van der Maaten L (2014). Accelerating t-SNE using tree-based algorithms. J. Mach. Learn. Res..

[79] Krijthe, J. H. Rtsne: t-Distributed Stochastic Neighbor Embedding using a Barnes-Hut implementation, version 0.13. (2015).

[80] Ester, M., Kriegel, H.-P., Sander, J. & Xu, X. A density-based algorithm for discovering clusters in large spatial databases with noise. *Proc. Second International Conference on Knowledge Discovery and Data Mining (KDD-96)* **96**, 226–231 (1996).

[81] Dunn JC (1974). Well-separated clusters and optimal fuzzy partitions. J. Cybern..

[82] Hahsler M, Piekenbrock M (2018). Dbscan: density based clustering of applications with noise (DBSCAN) and related algorithms. R. package version.

[83] Desgraupes, B. ClusterCrit: Clustering Indices. R package version 1.2.8. (2018).

[84] Robinson MD, McCarthy DJ, Smyth GK (2010). edgeR: a Bioconductor package for differential expression analysis of digital gene expression data. Bioinformatics.

[85] Langfelder P, Horvath S (2008). WGCNA: an R package for weighted correlation network analysis. BMC Bioinforma..

[86] Zhang, B. & Horvath, S. A general framework for weighted gene co-expression network analysis. *Stat. Appl. Genet. Mol*. **4**, 17 (2005). 10.2202/1544-6115.1128 16646834

[87] Supek F, Bošnjak M, Škunca N, Šmuc T (2011). REVIGO summarizes and visualizes long lists of gene ontology terms. PLoS ONE.

[88] Alexa A, Rahnenfuhrer J (2010). topGO: enrichment analysis for gene ontology. R. package version.

[89] Galili T, O’Callaghan A, Sidi J, Sievert C (2018). heatmaply: an R package for creating interactive cluster heatmaps for online publishing. Bioinformatics.

[90] Lenz, P. H. et al. Diapause vs. reproductive programs: transcriptional phenotypes in *Calanus finmarchicus*. *Dryad*, *Dataset*, 10.5061/dryad.12jm63xw7 (2021).

