# Supplementary material for: Diapause vs. reproductive programs: transcriptional phenotypes in a keystone copepod
Source: Commun Biol. 2021 Mar 29;4:426. doi: 10.1038/s42003-021-01946-0 (PMC8007741; doi:10.1038/s42003-021-01946-0)
Supplement: Supplementary file 1 — Supplementary Information [file 42003_2021_1946_MOESM1_ESM.pdf]

Supplementary

Table S1. Candidate biomarker genes that are differentially expressed based on developmental program. Selection was based on studies in other arthropods (insects, *Artemia* franciscana) and *Caenorhabditis elegans* and cross-referenced against reported gene expression differences between active and diapausing *Calanus finmarchicus* and *Calanus sinicus* collected from the field (Aruda et al. 2011; Ning et al. 2013; Unal et al. 2013). For each potential candidate gene, a description of its function, its reported pattern of expression and reference has been included. References include both transcriptomic and proteomic studies. *C. finmarchicus* transcripts have been searched in the reference transcriptome based on their annotation (E-value ≤ 10^{-10}). Bold: transcripts that were differentially expressed (general linear model, $p \leq 0.05$).

**Target genes**	**Function and potential role in diapause preparation**	**References**	***C. finmarchicus* transcript (NCBI Acc. n.)**
HSP21	A small heat shock protein (*ArHsp21*) was up-regulated in diapause-bound *Artemia* embryos but not in direct developing embryos	Qiu and MacRae, 2008	**No match found**
Phosphoenolpyruvate carboxylase kinase (PEPCK)	PEPCK catalyzes the rate-limiting process in gluconeogenesis (sugar production from other substrates). PECK is up-regulated prior and during diapause in an insect	Poelchau et al., 2013	**GAXK01051558**
Nitric Oxide synthase (NOS)	NOS produces nitric oxide, a secondary messenger in cellular signaling pathways. In some insects NO concentrations peak during the early phases of diapause	Lu et al., 2013	GAXK01070927<br>**GAXK01205654**<br>GAXK01001099<br>**GAXK01152094**<br>GAXK01117405<br>GAXK01193687<br>GAXK01151119<br>GAXK01136162
Serpins	Serpins inhibit and regulate activity of serine proteases. Some serpins exhibit high expression during diapause preparation in insects	Pérez-Hedo et al., 2012<br>Zhang et al., 2013	**GAXK01189184**<br>**GAXK01085717**<br>GAXK01201136<br>**GAXK01130390**
Ras-related protein Rab-10	“Rab” genes are GTPases that act as metabolic switches to regulate intracellular vesicle transport. Rab-10 is a key regulatory of autophagy pathways and is associated with longevity during dormancy (dauer) in *C. elegans*	Hansen et al., 2008	**GAXK01056549**

References

Aruda, A. M., M. F. Baumgartner, A. M. Reitzel, and A. M. Tarrant. 2011. Heat shock protein expression during stress and diapause in the marine copepod *Calanus finmarchicus*. J. Insect Physiol. **57:** 665-675.

Hansen, M., A. Chandra, L. L. Mitic, B. Onken, M. Driscoll, and C. Kenyon. 2008. A role for autophagy in the extension of lifespan by dietary restriction in *C. elegans*. PLoS Genetics **4**.

Lu, M.-X., S.-S. Cao, Y.-Z. Du, Z.-X. Liu, P. Liu, and J. Li. 2013. Diapause, signal and molecular characteristics of overwintering *Chilo suppressalis* (Insecta: Lepidoptera: Pyralidae). Sci. Rep. **3:** 1-9.

Ning, J., M. X. Wang, C. L. Li, and S. Sun. 2013. Transcriptome sequencing and *de novo* analysis of the copepod *Calanus sinicus* using 454 GS FLX. PloS One **8:** e63741.

Pérez-Hedo, M., I. Sánchez-López, and M. Eizaguirre. 2012. Comparative analysis of hemolymph proteome maps in diapausing and non-diapausing larvae of Sesamia nonagrioides. Proteome Sci. **10:** 58.

Poelchau, M. F., J. A. Reynolds, C. G. Elsik, D. L. Denlinger, and P. A. Armbruster. 2013. Deep sequencing reveals complex mechanisms of diapause preparation in the invasive mosquito, *Aedes albopictus*. Proc. R. Soc. B **280**.

Qiu, Z., and T. H. MacRae. 2008. ArHsp21, a developmentally regulated small heat-shock protein synthesized in diapausing embryos of *Artemia franciscana*. Biochem. J. **411:** 605-611.

Unal, E., A. Bucklin, P. H. Lenz, and D. W. Towle. 2013. Gene expression of the marine copepod *Calanus finmarchicus*: responses to small-scale environmental variation in the Gulf of Maine (NW Atlantic Ocean). J. Exp. Mar. Biol. Ecol. **446:** 76-85.

Zhang, Q., Y.-X. Lu, and W.-H. Xu. 2013. Proteomic and metabolomic profiles of larval hemolymph associated with diapause in the cotton bollworm, *Helicoverpa armigera*. BMC Genomics **14:** 751

Supplementary note: Methods and analysis to address the multi-species question

As described in the main text, the samples analyzed in this study, while originally thought to contain a single species of copepod, *Calanus finmarchicus*, also turned out to contain as many as two congers in some samples. Species identification of calanoid copepods is a well-known challenge. Congeners are morphologically very similar and often are only reliably identified to species using genetic tools (Bucklin et al. 2018). This is particularly true for the *Calanus* congeners that co-occur in the Norwegian Sea (Choquet et al. 2018). Thus, it is not surprising that a detailed genetic analysis of the field samples collected from Trondsheimfjord led to the identification of *C. glacialis* and *C. helgolandicus* in addition to the target species *C. finmarchicus*. However, the effect of this multiplicity on transcriptomic gene expression profiles is expected to be minor since the reference transcriptome is species specific (*C. finmarchicus*). Furthermore, all three species are on the diapause program in Trondsheimfjord in late May and June. This would lead to similar gene expression profiles in the congeners, which reduces the likely impact of congeneric contamination in the gene expression data.

Nevertheless, it is still useful to consider the potential effect of such species multiplicity. The genetic similarity among congeneric species in the samples leads to cross-mapping onto the reference by their short reads (Lenz et al. 2021), which potentially introduces variation to the gene expression analysis. The question then becomes whether the sample mixture in our data set

might have introduced a bias that has affected the results of the analysis. We conducted a series of tests and conclude that there is no systematic bias that could invalidate the results. This should not be surprising, since cross-mapping is inefficient. Interspecific sequence variations inhibit mapping success and limit the contribution of the foreign species to the gene expression data.

The first test focused on species composition in each sample to estimate contribution of each species to the short sequence reads generated by the RNA-Seq technology. For species identification and relative abundance in a sample we generated a “species reference” composed of three mtCOI sequences, one from each congener. Each RNA-Seq sample was mapped against this reference using bowtie2. We vetted this method for species composition determination using publicly available RNA-Seq data from a known pure *C. finmarchicus* sample originating from the Gulf of Maine (NCBI accession SRX450620). The short reads from these data mapped exclusively to the mtCOI sequence from *C. finmarchicus* with not a single read mapping to either one of the two congeners. In contrast, at least a few reads mapped to the two congener sequences in every Norwegian sample. The percentages of short reads that mapped to each varied from insignificant to >50% (supplementary note Table 1). Significant congener contamination was limited to the field samples. Those samples, most of which had under 32% congener representation, could be separated into four classes of contamination. In order of decreasing *C. finmarchicus* representation (supplementary note Figure 1A right panel) these were: 1) samples EF1, EF4 and LF1: 74-75% *C. finmarchicus* (orange squares); 2) samples EF2 and LF2: 68-70% (green diamonds); 3) sample EF3 and LF4: 47-50% (blue inverted triangles); sample LF3: 39% (violet triangle). In spite of this heterogeneity among the field samples, they consistently clustered together in the t-SNE plots, while the nearly homogeneous *C. finmarchicus* culture samples, both early and late, clustered into two separate groups. As we have shown previously, major differences in transcriptional phenotypes show up as distinct clustering in the t-SNE plots (Cieslak et al 2020). Thus, one would predict that the contaminated samples would cluster according to contamination level, however, this did not occur (supplementary note Figure 1A, right panel).

In the second test, we estimated the percentage of foreign reads that mapped to the *C. finmarchicus* reference transcriptome for each sample. Cross-mapping of short sequence reads of *Calanus* congeners against the *C. finmarchicus* reference is highly reduced -- around 30% relative to *C. finmarchicus* mapping rates (Lenz et al. 2021; 133,576,022 of 485,722,542 or 28% for the *C. helgolandicus* read set described below). Combined with the contamination levels in the different field samples described above, this indicated a modest 11% estimate for mean cross-mapping levels (range: 7.4 to 18% - see supplementary note Table 1). While not insignificant, an average of 11% contamination would probably not greatly influence our overall results even if the congeners were on an entirely different developmental program from *C. finmarchicus* -- provided that the average applies to the critical genes governing developmental program.

However, different genes evolve at different rates, and the distribution of cross-mapped reads is not expected to be uniform across all transcripts in the reference transcriptome. A disproportion in high vs low cross-mapping-prone transcripts in different groups of samples could result in an artifactual bias in assessing transcriptional phenotypes. Thus, to obtain an estimate of which transcripts were attracting significant numbers of reads from the two

congeners, we examined cross-mapping of reads obtained from publicly available RNA-Seq data sets (*C. helgolandicus*: SRR12052971; and *C. glacialis*: SRR5004099). After an initial review, we focused on *C. helgolandicus*, given the depth of sequencing (485M short reads) and low contamination of reads that mapped to rRNA transcripts (< 2%). Of the 96,090 *C. finmarchicus* reference transcripts, 56,915 (59%) attracted at least one *C. helgolandicus* cross-mapped read, and 13,038 (13.6%) received 486 or more reads (>1 cpm). The latter provided us with a target list of transcripts that are evolutionarily conserved in the *Calanus* genus and are prone to cross-mapping. A review of the pool of 27,870 transcripts that passed the requirements for statistical testing (see Methods) identified 45% (12,500) that were on the list of 13K >1 cpm cross-mapping-prone transcripts. To assess whether there was a disproportionate share (bias) of cross-mapping-prone transcripts among the various subsets of transcripts used in the down-stream analyses, this 45% was compared with the presence of cross-mapping-prone transcripts among the DEGs and other gene filters. As presented in supplementary note Table 2, the percentages were somewhat higher than in the original pool from which the transcript sets were drawn, but all were within 10 percentage points. While this is not insignificant, it would not suggest a great influence over our results. Evolutionarily conserved genes typically serve essential functions; thus it is not surprising that cross-mapping-prone transcripts were well represented among the DEGs and the enriched processes involved in basic functions such as metabolism, transcription and reproduction.

The "designer filters" had more elevated proportions of cross-mapping-prone transcripts. The highest percentage, and thus most susceptible to possible bias from congener contamination, was found among the 111 transcripts in the “reproductive program” filter (89%). Interestingly, this was one filter that failed to separate the field samples from the culture ones, combining all samples into a single and highly dispersed cluster. Strong bias from congener contamination should have separated this into field vs culture clusters. The second largest percentage of 63% was found for the 54 transcripts in the “diapause program” filter. It is harder to rule out bias in this case, but it is the filter with the strongest phenotypic distinction between reproductive program (culture) and diapause program (field). Overall, this analysis supports the conclusion of low impact from congener contamination.

A skeptic might still argue that the small enrichment in members of the 13K target list between the 29K statistical pool (45%) and the DEGs (49%), the GO functional filters (up to 54%) and the designer filters (up to 89%) are responsible for the results of the analysis. In a third test of this hypothesis, we eliminated all significantly cross-mapping-prone transcripts (cpm >1) and repeated the t-SNE analysis of Figures 2, 5 and 9 (main text). We compared the clustering produced by this reduced set of transcripts with that from the original sets. The t-SNE results are shown in supplementary note Figure 1 with the left column of plots (“All”) including all transcripts in a set and the right column (“Low cross-map”) excluding transcripts prone to cross mapping. The t-SNE results without cross-mapped reads are largely the same as those with the full transcript sets, generating between two and four clusters depending on the transcript filter. Despite the reduced data set membership in the clusters was consistent with a single exception: we found one late field sample (LF4, < 50% *C. finmarchicus*; arrow in supplementary note Figure 1B) that changed grouping using the oogenesis functional filter. A second difference was in the number of distinct clusters occurring in the plots. In three cases, oogenesis, RNA metabolic process and reproductive program, the removal of cross-mapped reads separated one

of the original clusters (in the "All" panel") into two. The oogenesis filter made a separate cluster out of the EC samples, which previously had been clustered with the field samples, enhancing, not decreasing, the transcriptional distinction between field and culture. The RNA metabolic process filter separated the culture samples into early and late, without affecting the separation between samples with high vs low species heterogeneity. The reproductive filter separated its single large group into two according to sample origin (field vs culture). In no cases did the elimination of cross-mapping-prone transcripts result in a merger of clusters, separation of which might have been ascribed to congener-derived bias. Thus, it would appear that correlated with the reduced number of cross-map-prone transcripts, a separation of the samples into distinct transcriptional phenotypes becomes more evident, rather than less so.

In summary, the comparisons between diapause and reproductive programs are robust even with the complicating factor of multiple species in the field samples. There is little evidence of systematic bias due to cross-mapping of short reads from the two congeners. While it would have been preferable to have avoided the contamination with congeners in the first place, the available dataset represents a unique opportunity that should not be lost. The research effort involved in generating these samples was substantial and not easily replicated. The functional analysis and interpretation of the data depended on the conserved genes, which are biologically essential justifying the inclusion of all transcripts in a comprehensive analysis. Excluding the conserved genes, which are cross-mapping-prone would have weakened the analysis.

Supplementary note Table 1. Summary of RNA-Seq dataset. RNA-Seq data for stage CV copepodids from 4 treatments (4 samples /treatment): Early field (EF), Late field (LF), Early culture (EC) and Late culture (LC) were downloaded from NCBI Bioproject PRJNA 231164. Sample IDs used in the publication and the treatment (source / time) are presented in the first three columns. Mapping percentage is the overall mapping rate of sequences that mapped to the Gulf of Maine *C. finmarchicus* transcriptome (96K transcripts, Lenz et al., 2014). Number of reads that aligned to species-specific mtCOI sequences for each species using bowtie2 (ambiguous mapping rate 1% or less). Rate gives the estimated proportion of *C. glacialis* and *C. helgolandicus* reads that contributed to reads that mapped to the *C. finmarchicus* reference ($0.3 \times (1-N_{Cfin}/N_{total})$). Last column provides NCBI accession numbers for the downloaded short sequence reads.

Sample ID	Treatment		% mapping	*C. finmarchicus*	*C. glacialis*	*C. helgolandicus*	Rate	Accession #
	Source	Time						
EF1	Field	Early	62.1	591,812	194,714	45	0.07	SRR1139735
EF2	Field	Early	60.9	503,631	218,843	27	0.09	SRR1139734
EF3	Field	Early	55.9	651,253	659,632	34	0.15	SRR1137919
EF4	Field	Early	60.9	574,678	204,345	23	0.08	SRR1138709
LF1	Field	Late	61.5	663,643	232,309	28	0.08	SRR1139728
LF2	Field	Late	59.2	510,727	103,657	133,070	0.10	SRR1139730
LF3	Field	Late	54.2	373,879	435,901	139,993	0.18	SRR1139732
LF4	Field	Late	55.2	422,199	256,728	211,179	0.16	SRR1139733
EC1	Culture	Early	68.5	783,160	1,013	789	0.001	SRR1141097
EC2	Culture	Early	68.2	940,188	137	42	<0.001	SRR1141098
EC3	Culture	Early	68.2	844,026	972	549	0.001	SRR1141099
EC4	Culture	Early	68.8	929,273	225	79	<0.001	SRR1141100
LC1	Culture	Late	78.0	434,829	79	9	<0.001	SRR1141107
LC2	Culture	Late	68.6	850,517	137	45	<0.001	SRR1141108
LC3	Culture	Late	66.0	689,218	194	43	<0.001	SRR1141109
LC4	Culture	Late	67.9	978,562	187	34	<0.001	SRR1141110

Supplementary note Table 2. Transcript analysis of cross-mapping of *C. helgolandicus* reads to the *C. finmarchicus* reference transcriptome. Lists of transcripts from different categories were analyzed for members that contributed to cross-mapping. Only transcripts with significant number of cross-mapped reads (>1 cpm [count per million]) were considered for this analysis.

Category	Transcripts in category (#)	Cross-mapped (#)	Percent (%)
EdgeR filter	27,870	12,500	44.9%
DEGs	11,503	5,625	48.9%
'fatty acid biosynthetic process' [GO:0006633]	70	38	54.3%
'RNA metabolic process'[GO:0016070]	1,064	545	51.2%
'oogenesis' [GO:0048477]	584	289	49.5%
'lipid metabolic process' [GO:0006629]	717	375	52.4%
Target genes	14	7	50.0%
Reproductive program filter	111	99	89.3%
Diapause program filter	54	34	63.0%

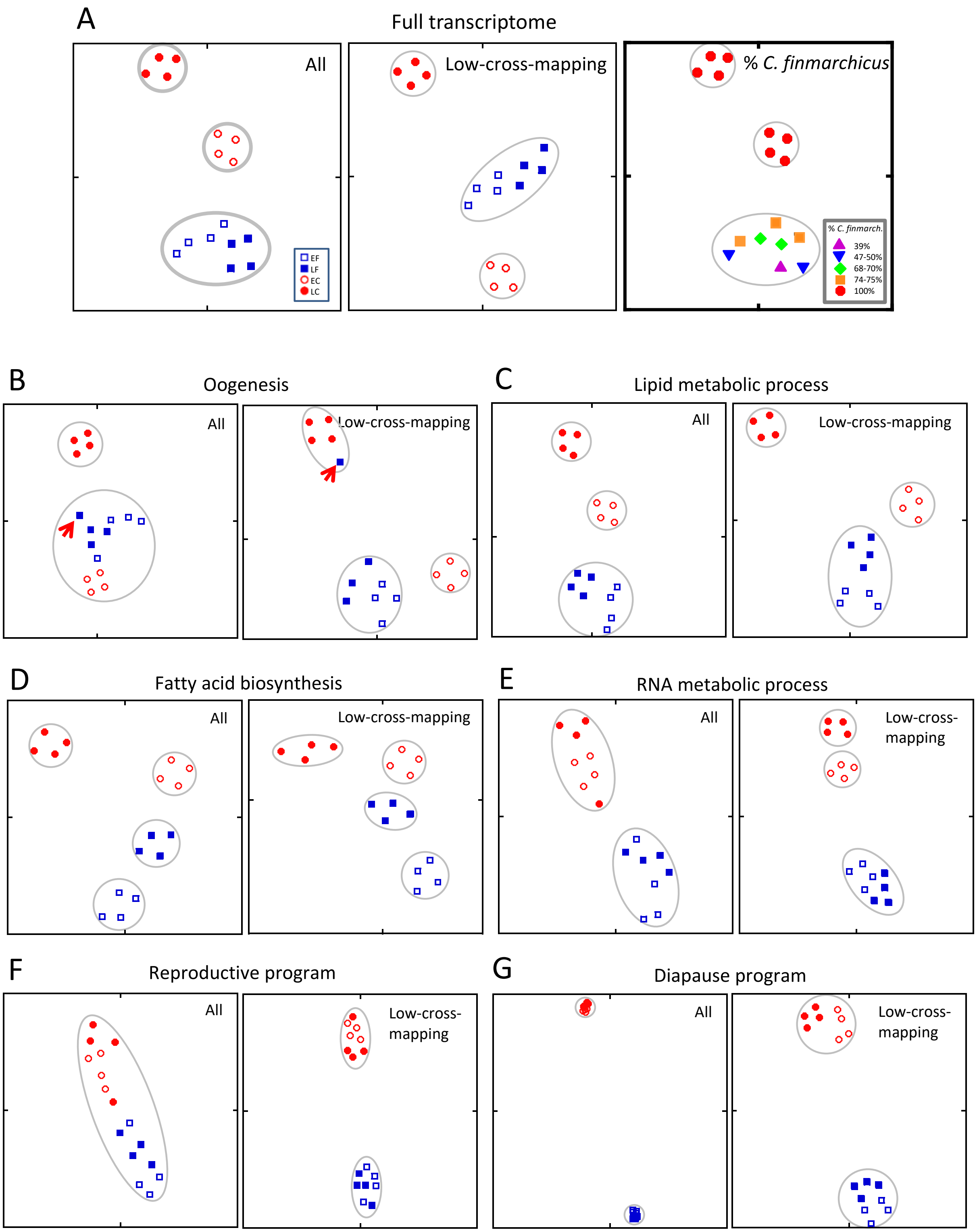

A
Full transcriptome
All
EF
LF
EC
LC
Low-cross-mapping
% C. finmarchicus
% C. finmarch.
39%
47-50%
68-70%
74-75%
100%
B
Oogenesis
All
Low-cross-mapping
C
Lipid metabolic process
All
Low-cross-mapping
D
Fatty acid biosynthesis
All
Low-cross-mapping
E
RNA metabolic process
All
Low-cross-mapping
F
Reproductive program
All
Low-cross-mapping
G
Diapause program
All
Low-cross-mapping

Supplementary note Figure 1.
t-SNE plots for subsets of transcripts filtered according to membership in different gene ontology (GO) terms and their child terms from Figures 5 and 9, main text. Circular profiles enclose clusters as determined by DBSCAN algorithm (if not obvious). Left panels ("All") contain the entire filter dataset; right panels ("Low cross-mapping") contain only those transcripts with cpm<1 for mapping of *C. helgolandicus* reads. A. Entire 96K reference transcriptome; the third panel parses the samples according to % reads mapped to mtCOI sequence for *C. finmarchicus* vs to congeners. B. ‘oogenesis’ filter (GO:0048477)(Dunn$_{LoX}$: 1.292, 2 clusters); arrow points to LF4 sample; C. ‘lipid metabolic process’ filter (GO:0006629)(Dunn$_{LoX}$=0.700, 4 clusters, eps=6.5); DunnLoX=0.587 3 clusters; D. ‘fatty-acid biosynthesis’ filter (GO:0006633)(Dunn$_{LoX}$=0.676;=0.620; 3 clusters; E. ‘RNA metabolic process’ filter (GO:016070)(Dunn$_{cross}$=0.628 with 3 clusters). F. Reproductive program "Filter 5" (Figure 9C)(Dunn$_{LoX}$=2.73) ; G. Diapause program "Filter 4" (Figure 9E)(Dunn$_{LoX}$=3.055). For the full transcript sets ("All") only the RNA filter and Filter 4 divided the samples into separate field and culture transcriptional phenotypes. For the low cross-map set, Filter 5 (Reproductive) made a clean separation as well, but it contained only 12 transcripts, which is too few to be given much weight. Symbol coding: field samples: squares; culture samples: circles; early samples open symbols; late samples: closed symbols. All panels: perplexity = 5; number of iterations = 50,000 [A: 2021-02-02 R5 Oogenesis dups removed B-D: 2019-12-19 runs]

Supplementary note References

Asai, S., Sanges, R., Lauritano, C., Lindeque, P.K., Esposito, F., Ianora, A., Carotenuto Y. (2020) *De novo* transcriptome assembly and gene expression profiling of the copepod *Calanus helgolandicus* feeding on the PUA-producing diatom *Skeletonema marinoi*. *Marine drugs,* **18**,392.

Bucklin, A., Divito, K. R., Smolina, I., Choquet, M., Questel, J. M., Hoarau, G. and O'neill, R. J. (2018) Population genomics of marine zooplankton. In: O. M. Rajora and M. Oleksiak (eds) *Population Genomics: Marine Organisms.* Springer, pp. 61-102.

Choquet, M., Kosobokova, K., Kwaśniewski, S., Hatlebakk, M., Dhanasiri, A. K., Melle, W., Daase, M., Svensen, C., Søreide, J. E. and Hoarau, G. (2018) Can morphology reliably distinguish between the copepods *Calanus finmarchicus* and *C. glacialis*, or is DNA the only way? *Limnol Oceanogr: Methods,* **16,** 237-252.

Lenz, P. H., Roncalli, V., Hassett, R. P., Wu, L. S., Cieslak, M. C., Hartline, D. K. and Christie, A. E. (2014) *De novo* assembly of a transcriptome for *Calanus finmarchicus* (Crustacea, Copepoda)--the dominant zooplankter of the North Atlantic Ocean. *PloS One,* **9,** e88589.

Lenz, P. H., Lieberman, B., Cieslak, M. C., Roncalli, V. and Hartline, D. K. (2021) Transcriptomics and metatranscriptomics in zooplankton: wave of the future? *J Plankton Res*., **43(1),** 3–9.
