# Supplementary material for: Diapause vs. reproductive programs: transcriptional phenotypes in a keystone copepod
Source: Commun Biol. 2021 Mar 29;4:426. doi: 10.1038/s42003-021-01946-0 (PMC8007741; doi:10.1038/s42003-021-01946-0)
Supplement: Supplementary file 2 — Reporting Summary [file 42003_2021_1946_MOESM2_ESM.pdf]

nature research

Corresponding author(s): Vittoria Roncalli

Last updated by author(s): Feb 24, 2021

Reporting Summary

Nature Research wishes to improve the reproducibility of the work that we publish. This form provides structure for consistency and transparency in reporting. For further information on Nature Research policies, see our Editorial Policies and the Editorial Policy Checklist.

Statistics

For all statistical analyses, confirm that the following items are present in the figure legend, table legend, main text, or Methods section.

n/a	Confirmed	
☐	☒	The exact sample size (n) for each experimental group/condition, given as a discrete number and unit of measurement
☐	☒	A statement on whether measurements were taken from distinct samples or whether the same sample was measured repeatedly
☐	☒	The statistical test(s) used AND whether they are one- or two-sided<br>*Only common tests should be described solely by name; describe more complex techniques in the Methods section.*
☒	☐	A description of all covariates tested
☒	☐	A description of any assumptions or corrections, such as tests of normality and adjustment for multiple comparisons
☒	☐	A full description of the statistical parameters including central tendency (e.g. means) or other basic estimates (e.g. regression coefficient) AND variation (e.g. standard deviation) or associated estimates of uncertainty (e.g. confidence intervals)
☒	☐	For null hypothesis testing, the test statistic (e.g. F, t, r) with confidence intervals, effect sizes, degrees of freedom and P value noted<br>*Give P values as exact values whenever suitable.*
☒	☐	For Bayesian analysis, information on the choice of priors and Markov chain Monte Carlo settings
☒	☐	For hierarchical and complex designs, identification of the appropriate level for tests and full reporting of outcomes
☒	☐	Estimates of effect sizes (e.g. Cohen's d, Pearson's r), indicating how they were calculated

Our web collection on statistics for biologists contains articles on many of the points above.

Software and code

Policy information about availability of computer code

Data collection	No software were used during collection
Data analysis	Software used for dpownstream analyses: Bowtie2 (v.2.1.0), ReviGO (http://revigo.irb.hr/), AmiGO software GO Online SQL Environment (GOOSE). R packages: Rtsne, edgeR, WGCNA, topGO (v. 2.88.0), heatmaply.

For manuscripts utilizing custom algorithms or software that are central to the research but not yet described in published literature, software must be made available to editors and reviewers. We strongly encourage code deposition in a community repository (e.g. GitHub). See the Nature Research guidelines for submitting code & software for further information.

Data

Policy information about availability of data

All manuscripts must include a data availability statement. This statement should provide the following information, where applicable:

- Accession codes, unique identifiers, or web links for publicly available datasets
- A list of figures that have associated raw data
- A description of any restrictions on data availability

The study uses a previously generated dataset publicly available on the National Center for Biotechnology Information (NCBI) database (BioProject: PRJNA 352 231164). Short-read sequences for 16 samples were downloaded from the short-sequence read archive (SRA) and mapped against an existing Calanus finmarchicus transcriptome also available on NCBI (Bioproject PRJNA236528). Additional files used for downstream analyses have been deposited on DryAd dataset (doi:https://doi.org/10.5061/dryad.12jm63xw7).

Field-specific reporting

Please select the one below that is the best fit for your research. If you are not sure, read the appropriate sections before making your selection.

☐ Life sciences ☐ Behavioural & social sciences ☒ Ecological, evolutionary & environmental sciences

For a reference copy of the document with all sections, see nature.com/documents/nr-reporting-summary-flat.pdf

Ecological, evolutionary & environmental sciences study design

All studies must disclose on these points even when the disclosure is negative.

Study description	The study used an existing RNA-Seq dataset generated by Tarrant and colleagues (Tarrant et al., 2014, 2016) for the calanoid copepod Calanus finmarchicus pre-adult copepodids (CV). The dataset included a laboratory-cultured population and a field-collected wild with two time-points each (early and late). The two populations could be distinguished, based on the developmental programs; the laboratory-cultured group was on the "reproductive program" while the field-collected group was on the "diapause program". Briefly, the laboratory-cultured samples consisted of recently molted (≤ 24 hrs) stage CV copepodids that had been isolated and incubated separately until harvested at three (early culture, "EC") and 10 days (late culture, "LC") post-molt. The goal was to determine whether the two programs could be separated by their respective gene expression (transcriptomic) phenotypes, and whether this difference would lead to new insights into the physiological basis of the diapause program.
Research sample	The calanoid copepod Calanus finmarchicus is key stone species in the North Atlantic. In the North Atlantic, the copepod Calanus finmarchicus completes one to three generations annually with some individuals maturing into adults, while others interrupt their development to enter diapause. It is unknown which, why and when individuals enter the diapause program. The study focuses on pre-adults (CV).
Sampling strategy	Field samples were collected from Trollet Station in Trondheimsfjord, Norway) on May 28, 2013 (early field, "EF") and 14 days later on June 10, 2013 (late field, "LF). Additional details on the experiments can be found in previous studies: Tarrant et al., 2014 and 2016.
Data collection	Additional details on the sampling can be found in Tarrant et al., 2014 and 2016.
Timing and spatial scale	Additional details on the sampling can be found in Tarrant et al., 2014 and 2016.
Data exclusions	No data were excluded
Reproducibility	Our study uses a previouly generated dataset. The goal of the study
Randomization	Randomization was not used in this study considering the experimnetal design.
Blinding	Blinding was not relevant to this study, considering the experimnetal design.

Did the study involve field work? ☐ Yes ☒ No

Reporting for specific materials, systems and methods

We require information from authors about some types of materials, experimental systems and methods used in many studies. Here, indicate whether each material, system or method listed is relevant to your study. If you are not sure if a list item applies to your research, read the appropriate section before selecting a response.

Materials & experimental systems

n/a	Involved in the study
☒	☐ Antibodies
☒	☐ Eukaryotic cell lines
☒	☐ Palaeontology and archaeology
☒	☐ Animals and other organisms
☒	☐ Human research participants
☒	☐ Clinical data
☒	☐ Dual use research of concern

Methods

n/a	Involved in the study
☒	☐ ChIP-seq
☒	☐ Flow cytometry
☒	☐ MRI-based neuroimaging
